# An operon consisting of a P-type ATPase gene and a transcriptional regulator gene responsible for cadmium resistances in *Bacillus vietamensis* 151–6 and *Bacillus marisflavi* 151–25

**DOI:** 10.1186/s12866-020-1705-2

**Published:** 2020-01-21

**Authors:** Xiaoxia Yu, Zundan Ding, Yangyang Ji, Jintong Zhao, Xiaoqing Liu, Jian Tian, Ningfeng Wu, Yunliu Fan

**Affiliations:** 1 Biotechnology Research Institute, Chinese Academy of Agricultural Sciences, Beijing, China; 2 College of Life Science and Technology, Jinan University, Guangzhou, Guangdong China

**Keywords:** Cadmium resistance, Genomic sequencing, *Bacillus vietamensis* 151–6, *Bacillus marisflavi* 151–25

## Abstract

**Background:**

Cadmium (Cd) is a severely toxic heavy metal to most microorganisms. Many bacteria have developed Cd^2+^ resistance.

**Results:**

In this study, we isolated two different Cd^2+^ resistance *Bacillus* sp*.* strains, *Bacillus vietamensis* 151–6 and *Bacillus marisflavi* 151–25, which could be grown in the presence of Cd^2+^ at concentration up to 0.3 mM and 0.8 mM, respectively. According to the genomic sequencing, transcriptome analysis under cadmium stress, and other related experiments, a gene cluster in plasmid p25 was found to be a major contributor to Cd^2+^ resistance in *B. marisflavi* 151–25. The cluster in p25 contained *orf4802* and *orf4803* which encodes an ATPase transporter and a transcriptional regulator protein, respectively. Although 151–6 has much lower Cd^2+^ resistance than 151–25, they contained similar gene cluster, but in different locations. A gene cluster on the chromosome containing *orf4111*, *orf4112* and *orf4113*, which encodes an ATPase transporter, a cadmium efflux system accessory protein and a cadmium resistance protein, respectively, was found to play a major role on the Cd^2+^ resistance for *B. vietamensis* 151–6.

**Conclusions:**

This work described cadmium resistance mechanisms in newly isolated *Bacillus vietamensis* 151–6 and *Bacillus marisflavi* 151–25. Based on homologies to the *cad* system (CadA-CadC) in *Staphylococcus aureus* and analysis of transcriptome under Cd^2+^ induction, we inferred that the mechanisms of cadmium resistance in *B. marisflavi* 151–25 was as same as the *cad* system in *S. aureus*. Although *Bacillus vietamensis* 151–6 also had the similar gene cluster to *B. marisflavi* 151–25 and *S. aureus*, its transcriptional regulatory mechanism of cadmium resistance was not same. This study explored the cadmium resistance mechanism for *B. vietamensis* 151–6 and *B. marisflavi* 151–25 and has expanded our understanding of the biological effects of cadmium.

## Background

Soil contamination with heavy metals is becoming an increasingly urgent problem worldwide. Among all heavy metals, one of the most hazardous is cadmium (Cd) [1, 2]. Cd is a non-essential heavy metal element that is toxic, teratogenic and carcinogenic to humans. When critical Cd levels in soil are reached, biodiversity, agricultural productivity, food safety and human heath can be threatened, as Cd can be accumulated in the food chain [3–7]. Consequently, solutions to remediate heavy metal-contaminated soil, in particular Cd-contaminated soil, are urgently needed. Many remediation techniques, such as chemical immobilization, electrokinetic extraction, phytoremediation, and bioremediation, have been proposed for soils contaminated with heavy metals [8]. Among these technologies, bioremediation is considered an innovative and promising remediation method based on its cost effectiveness and low environmental impact [9–11].

Many organisms have developed strategies to withstand the presence of Cd in the environment, such as exclusion, compartmentalization, deployment of inorganic polyphosphates, cell wall binding, and expression of metal binding proteins [12–16]. The most prominent mechanism of resistance to Cd^2+^ is the use of efflux pumps [17]. Several bacteria, such as *Alcaligenes eutriphus* [18]*, Staphylococcus aureus* [19], *Bacillus subtilis* [20], *Listeria monocytogenes* [21] and *Escherichia coli* [22] have demonstrated resistance to Cd^2+^.

One of the most thoroughly characterized Cd^2+^ resistance efflux systems is the czc (Cd^2+^, Zn^2+^, and Co^2+^ resistance) system in the gram-negative bacterium *Alcaligenes eutrophus* [18, 23]. The CzcA, CzcB and CzcC proteins comprise an active efflux pump complex driven by a cation-proton antiporter [18]. Briefly, CzcA, located in the inner membrane, is essential for cation transport and can remove heavy metals (Cd^2+^, Zn^2+^, and Co^2+^) from the cytoplasm using a H^+^ ion gradient [23, 24]. CzcB belongs to a family of bacterial membrane fusion proteins. It may create a pathway for the removal of cations from CzcA [23, 25]. CzcC relies on CzcB to function and may act as a substrate (Cd^2+^, Zn^2+^, and Co^2+^) switch for the efflux pump [17].

Another well-characterized Cd^2+^ resistance system is the *cad* system (*cadA-cadC*) in the gram-positive bacterium *S. aureus* [19, 26]. The Cd-efflux ATPase is encoded by the *cadA* gene, which contains six predicted membrane-spanning regions. The fourth membrane span is thought to be involved in the cation translocation pathway, and includes a conserved Cys-Pro-Cys tripeptide [27]. CadC is a regulatory protein encoded immediately downstream of *cadA*, and is also required for Cd^2+^ resistance in *S. aureus* [28]. CadC is a member of the ArsR/SmtB family [29, 30], which can bind to the promoter-operator area of the *cadA-cadC* system and acts as a transcriptional repressor in vitro [31].

In present study, two different Cd^2+^ resistance *Bacillus* sp*.* strains (*B. vietamensis* 151–6 and *B. marisflavi* 151–25) isolated from Cd-contaminated soil was grown in the presence of Cd^2+^ at two distinct concentrations up to 0.3 mM and 0.8 mM, respectively. In order to compare the mechanism of Cd^2+^ resistance for these two strains, their genome and transcriptome under Cd^2+^ stress were analyzed. Moreover, a fosmid library from genomic DNA of *B. vietamensis* 151–6 was constructed to further delineate the Cd^2+^ resistance-related genes. The functions of these genes were analyzed and verified through overexpression in *E. coli* and *B. subtilis*. The gene clusters of 4802–4803 in the plasmid p25 of *B. marisflavi* 151–25 and 4111–4112-4113 on the chromosome of *B. vietamensis* 151–6 played the major role on the Cd^2+^ resistance.

## Results

### Strain 151–6 and 151–25 exhibit different Cd^2+^ resistances

To isolate Cd-resistant bacteria, a Cd-contaminated (59.561 ± 3.76 mg Cd kg^− 1^) soil sample was collected in Hunan Province. A total of 21 strains of bacteria were isolated using LB agar plates containing 0.5, 1 and 2 mM Cd^2+^. The MIC-Cd values of those isolates were determined and ranged from 0.4 to 1.0 mM (Additional file 3: Table S3). Among them, the strains 151–25 and 151–6, which showed the highest and lowest level of Cd^2+^ resistance respectively, were selected for further study (Additional file 3: Table S3), and identified as *Bacillus* sp. through 16S rDNA analysis.

**Fig. 1:**
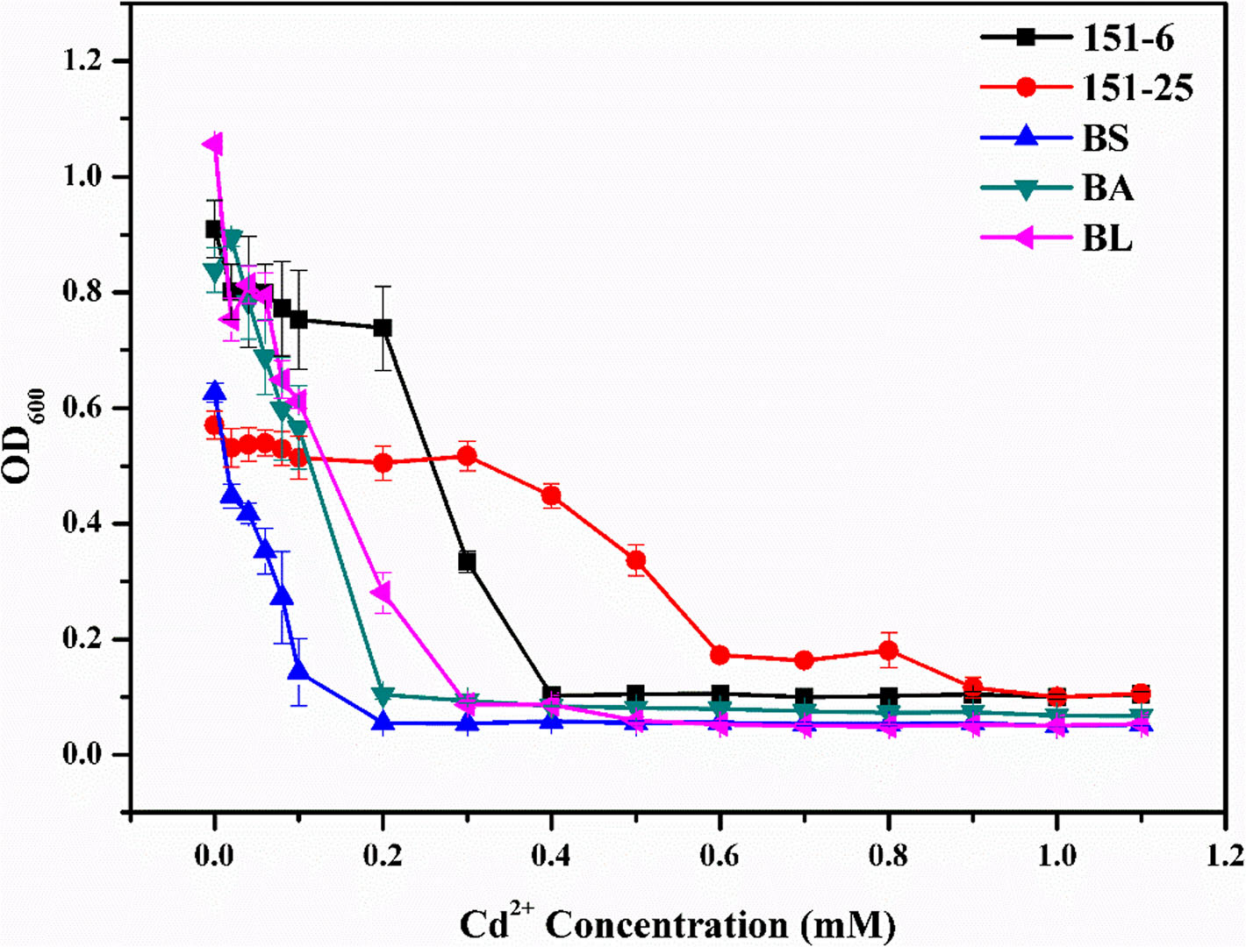
Evaluation of minimum inhibitory concentration of Cd^2+^ (MIC-Cd) for *Bacillus sp.* strains (151–6, 151–25, *B. subtilis* WB600 (BS), *B. amyloliquefaciens* (BA) and *B. licheniformis* WX-02 (BL)) at varying concentrations of Cd^2+^ (0, 0.02, 0.04, 0.06, 0.08, 0.1, 0.2, 0.3, 0.4, 0.5, 0.6, 0.7, 0.8, 0.9, 1.0 and 1.1 mM)

To compare the level of Cd^2+^ resistance of two selected strains with other *Bacillus* sp. bacteria, we examined their MIC-Cd, as well as those of three other *Bacillus* sp. strains, *B. subtilis* WB600 (BS), *B. amyloliquefaciens* (BA) and *B. licheniformis* WX-02 (BL), in liquid medium supplemented with various Cd^2+^ concentrations. 151–25 and 151–6 were grown in the presence of Cd^2+^ at concentrations up to 0.8 mM and 0.3 mM, respectively (Fig. 1), while the growth of the other three *Bacillus* sp. strains was inhibited in the presence of 0.3 mM Cd^2+^ (Fig. 1). The growth curves at 0 mM Cd^2+^ demonstrated that all four *Bacillus* sp. strains exhibited similar growth trends in liquid LB medium (Additional file 6: Figure S1a). However, under culture conditions with 0.1 mM or 0.3 mM Cd^2+^, 151–6 and 151–25 exhibited greater growth potential than the other three *Bacillus* sp. strains (Additional file 6: Figure S1b and c). And only 151–25 could grow at 0.5 mM Cd^2+^ (Additional file 6: Figure S1d). These data indicated that compared to other *Bacillus* sp. strains, 151–6 and 151–25 exhibited stronger tolerance to Cd^2+^. And the Cd^2+^ resistance of 151–25 was significantly higher than 151–6.

### Genomic analysis of 151–6 and 151–25

To compare the mechanism of Cd^2+^ resistance for 151–6 and 151–25, their genome were sequenced. The results showed that 151–6 contains a single chromosome (4,556,861 bp and 4952 predicted genes) and one plasmid, designated p6 (40,946 bp and 66 predicted genes) (Additional file 7: Figure S2). And 151–25 also contains a single chromosome (4,411,234 bp and 4734 predicted genes) and one plasmid, designated p25 (138,020 bp and 156 predicted genes) (Additional file 8: Figure S3). Simultaneously, the results of non-redundant protein database (NR) annotation of the predicted coding genes presented that strain 151–6 can be identified as *B. vietamensis* (4093 of the 5018 genes are annotated to derive from *B. vietamensis*) and strain 151–25 canbe identified as *B. marisflavi* (4095 of the 4890 genes are annotated to derive from *B. vietamensis*).

### Plasmid p25 in *B. marisflavi* 151–25 is important for Cd^2+^ resistance, while p6 in *B. vietamensis* 151–6 is not relevant to the Cd^2+^ resistance

**Fig. 2:**
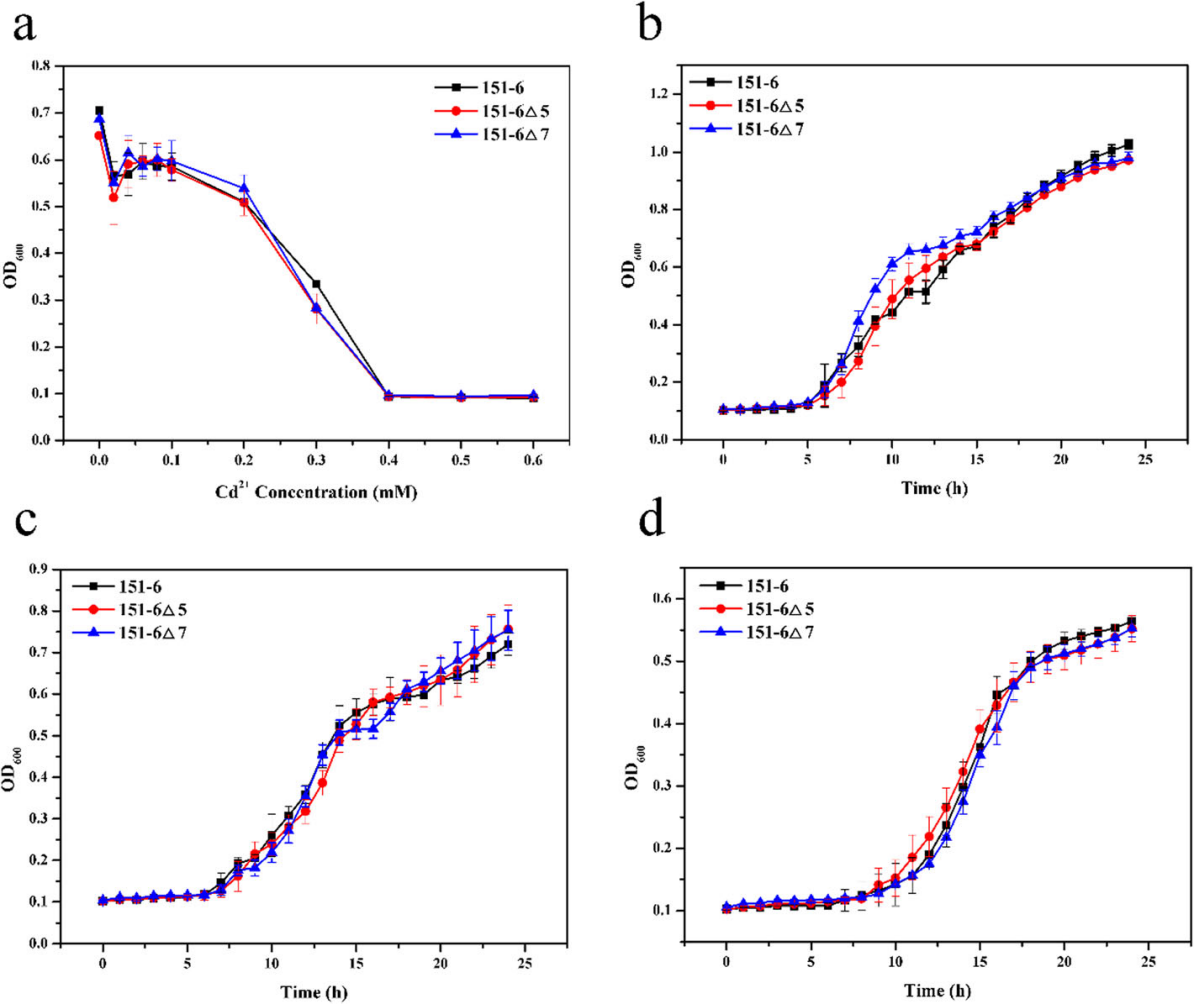
MIC-Cd and growth curves for the plasmid p6-eliminated strains 151–6△5, 151–6△7and wild strain *B. vietamensis* 151–6. **a** Evaluation of MIC-Cd for 151–6△5, 151–6△7and 151–6 at varying concentrations of Cd^2+^ (0, 0.02, 0.04, 0.06, 0.08, 0.1, 0.2, 0.3, 0.4, 0.5 and 0.6 mM). **b**, **c** and **d** Growth curve for 151–6△5, 151–6△7and 151–6 at various concentrations of Cd^2+^. (b) 0 mM Cd^2+^ added. **c** 0.1 mM Cd^2+^ added. (d) 0.2 mM Cd^2+^ added

**Fig. 3:**
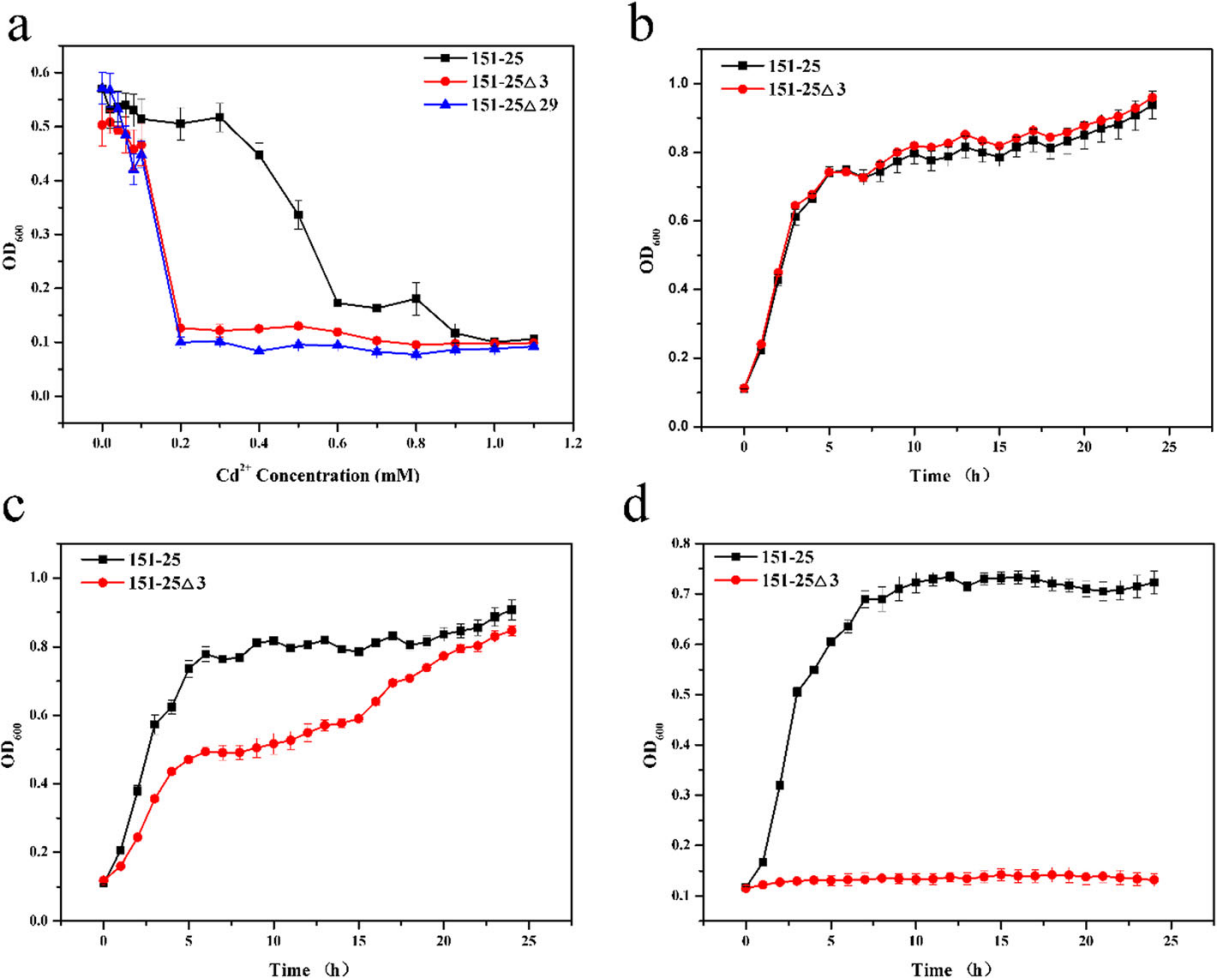
MIC-Cd and growth curves for the plasmid p25-eliminated strains 151–25△3 and 151–25△29, and the wild-type strain *B. marisflavi* 151–25. **a** Evaluation of MIC-Cd for 151–25△3, 151–25△29 and 151–25 at varying concentrations of Cd^2+^ (0, 0.02, 0.04, 0.06, 0.08, 0.1, 0.2, 0.3, 0.4, 0.5, 0.6, 0.7, 0.8, 0.9, 1.0 and 1.1 mM). **b**, **c** and **d**) Growth curves for 151–25△3, 151–25△29 and 151–25 at various concentrations of Cd^2+^. **b** 0 mM Cd^2+^ added. **c** 0.1 mM Cd^2+^ added. **d** 0.5 mM Cd^2+^ added

To determine whether plasmid p6 and p25 were responsible for Cd^2+^ resistance in *B. vietamensis* 151–6 and *B. marisflavi* 151–25, we eliminated the plasmids and then examined their contribution to Cd^2+^ resistance. The plasmid p6-eliminated strains 151–6△5 and 151–6△7 and the plasmid p25-deleted strains 151–25△3 and 151–25△29 were obtained (Additional file 9: Figure S4a and Additional file 10: Figure S5a, respectively) and further verified by PCR (Additional file 9: Figure S4b and Additional file 10: Figure S5b, 5c, respectively). In liquid media, the strains 151–6△5 and 151–6△7 exhibited the same MIC-Cd as the wild strain 151–6 (Fig. 2a). And the growth curve of 151–6△5 and 151–6△7 did not show any differences with wild type 151–6 at the three different concentrations of Cd^2+^ (Fig. 2b, c and d). These data indicated that the elimination of plasmid p6 does not influence the Cd^2+^ resistance of *B. vietamensis* 151–6. However, eliminating plasmid p25 caused a significant decreased of Cd^2+^ resistance in *B. marisflavi* 151–25. As shown in Fig. 3a, the MIC-Cd values of 151–25△3 and 151–25△29 were 0.2 mM, markedly lower than that of wild-type 151–25 (1.0 mM). Elimination of plasmid p25 did not affect the growth of the strain in liquid LB medium without added Cd^2+^ (Fig. 3b). However, with addition of 0.1 or 0.5 mM Cd^2+^ to the medium, the growth of 151–25△3 was inhibited, while the growth of *B. marisflavi* 151–25 was not affected (Fig. 3c and d, respectively). Thus, we concluded that plasmid p25 is important for Cd^2+^ resistance in *B. marisflavi* 151–25 while plasmid p6 is not relevant to the Cd^2+^ resistance in *B. vietamensis* 151–6.

### A gene cluster consisting of *orf4802* and *orf4803* confers high Cd^2+^ resistance in *B. marisflavi* 151–25

**Fig. 4:**
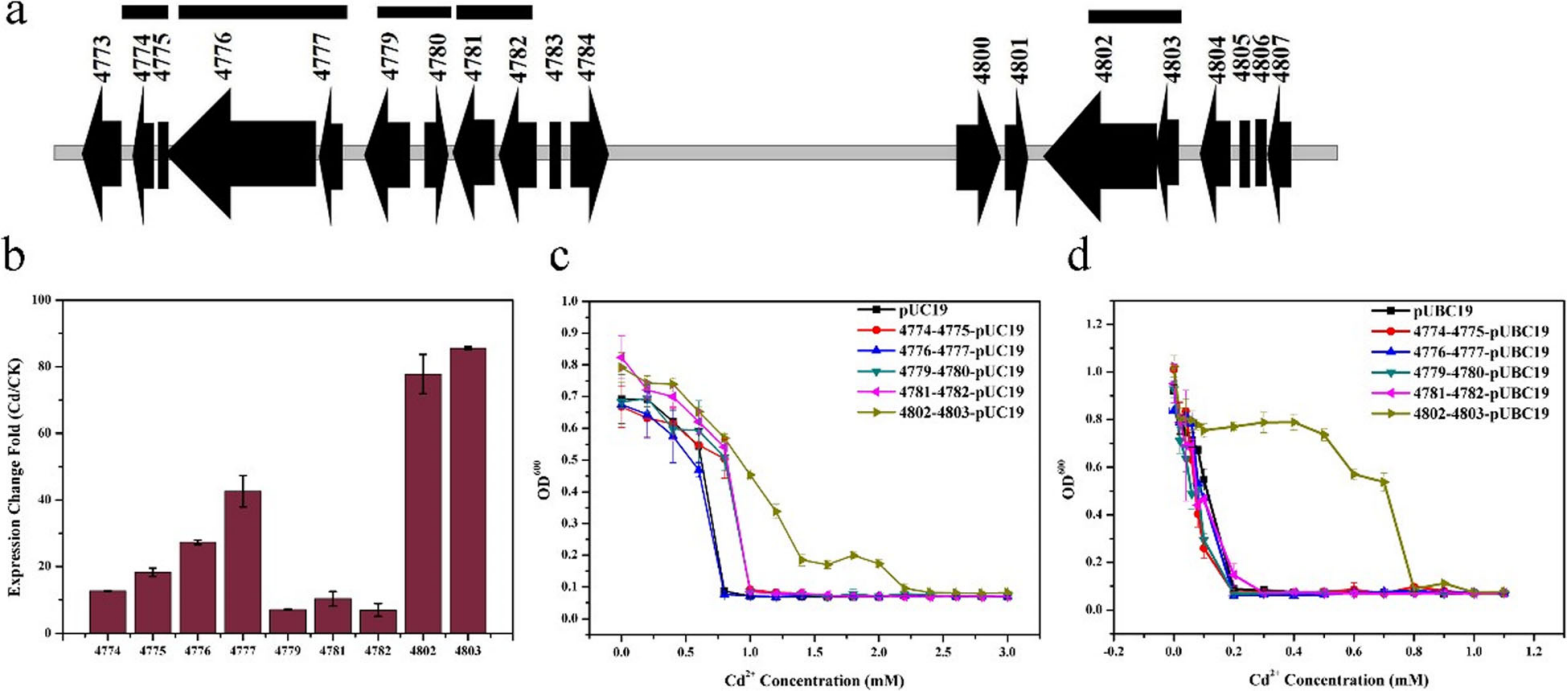
Genetic organization and transcription analysis of up-regulated genes based on RNA sequencing and examination of MIC-Cd for recombinant *E. coli* and *B. subtilis* containing the gene fragments of p25. **a** Schematic representation of loci n the plasmid p25 fragment. Thick gray line, plasmid p25 DNA; filled arrows and squares, 19 open reading frames with corresponding gene sizes; black horizontal line, overexpressed gene fragments. **b** Transcription analysis of the plasmid p25 genes *orf4774*, *4775*, *4776*, *4777*, *4779*, *4781*, *4782*, *4802* and *4803* in *B. marisflavi* 151–25 cultured with 0.1 mM Cd^2+^ − Cd in comparison with a culture grown in the absence of Cd^2+^ (CK) using quantitative reverse transcription-polymerase chain reaction (qRT-PCR). Transcript levels of tested genes were normalized to the 16S rRNA gene. **c** Determination of MIC-Cd for recombinant *E. coli* containing the vectors pUC19 (negative control), 4774–4775-pUC19, 4776–4777-pUC19, 4779–4780-pUC19, 4781–4782-pUC19 and 4802–4803-pUC19 with varying concentrations of Cd^2+^ (0, 0.2, 0.4, 0.6, 0.8, 1.0, 1.2, 1.4, 1.6, 1.8, 2.0, 2.2, 2.4, 2.6, 2.8 and 3.0 mM). **d** Determination of MIC-Cd for recombinant *B. subtilis* containing the vectors pUBC19 (negative control), 4774–4775-pUBC19, 4776–4777-pUBC19, 4779–4780-pUBC19, 4781–4782-pUBC19 and 4802–4803-pUBC19 with varying concentrations of Cd^2+^ (0, 0.02, 0.04, 0.06, 0.08, 0.1, 0.2, 0.3, 0.4, 0.5, 0.6, 0.7, 0.8, 0.9, 1.0 and 1.1 mM)

To determine the genes related to the Cd^2+^ resistance of *B. marisflavi* 151–25, its transcriptome under Cd^2+^ induction was analyzed by RNA sequencing. Fragments per kilobase of exon model per million mapped reads values were used to measure transcript abundance. As shown in Additional file 4: Table S4 and Additional file 11: Figure S6, a total of 65 differentially expressed genes were identified in the presence of Cd^2+^ (47 up-regulated genes and 18 down-regulated genes). Among the 47 up-regulated genes, 9 were located on plasmid p25, and the top three up-regulated genes in terms of fold change were also from plasmid p25 (Table 1 and Additional file 4: Table S4). Briefly, transcripts of a transcriptional regulator gene (*orf4803*) and a copper-translocating P-type ATPase gene (*orf4802*) were increased 72.53- and 63.83-fold, respectively. These results were confirmed through qRT-PCR (Fig. 4b), and it further indicated that plasmid p25 is related to Cd^2+^ resistance in *B. marisflavi* 151–25. Based on these results, we hypothesize that nine up-regulated genes located on plasmid p25, *orf4774*, *4775*, *4776*, *4777*, *4779*, *4781*, *4782*, *4802* and *4803* may be involved in Cd^2+^ resistance of *B. marisflavi* 151–25. To confirm this hypothesis, we overexpressed these genes using their own promoter with five gene clusters (Fig. 4a) in *E. coli* and *B. subtilis*, to examine their contributions to Cd^2+^ resistance. As shown in Fig. 4c and d, the gene cluster containing *orf4802* and o*rf4803* allowed recombinant *E. coli* and *B. subtilis* to exhibit higher Cd^2+^ resistance than negative control strains. In particular, *B. subtilis* cells containing the recombinant plasmid 4802–4803-pUBC19 would grow in the presence of Cd^2+^ at seven times the concentration tolerated by control cells. However, compared to control cells, the other four recombinant *E. coli* and *B. subtilis* strains did not show elevated Cd^2+^ resistance. These results indicate that the gene cluster consisting of *orf4802* and *orf4803* confers high Cd^2+^ resistance in *B. marisflavi* 151–25.

**Table 1:** Transcriptome annotation of up-regulated genes from the plasmid p25 of *B. marisflavi* 151–25

Gene	Location		Reads count		Fold change (Cd/CK)	NR annotation
	Start	End	Cd	CK		
*orf4803*	51,464	51,832	500.24	6.90	72.53	transcriptional regulator
*orf4802*	49,342	51,471	7460.73	116.89	63.83	copper-translocating P-type ATPase
*orf4775*	26,102	26,236	70.79	1.65	42.98	NA^a^
*orf4777*	28,766	29,110	45.73	1.75	26.10	putative transcriptional regulator
*orf4776*	26,251	28,752	3137.19	132.92	23.60	ATPase
*orf4774*	25,655	25,972	566.50	26.48	21.40	restriction endonuclease
*orf4782*	31,679	32,254	899.96	76.98	11.69	oxidoreductase
*orf4781*	31,185	31,682	619.15	54.15	11.43	oxidoreductase
*orf4779*	29,538	30,608	1066.87	220.52	4.84	arsenic resistance protein

^a^ No annotation information in NR database

To find more genes responsible for high Cd^2+^ resistance of *B. marisflavi* 151–25, we also compared gene expression between *B. marisflavi* 151–25 and 151–25△3 under Cd^2+^ stress. Nineteen up-regulated genes located on the chromosome that exhibited a more than 4-fold change according to transcriptome data were analyzed using qRT-PCR. Compared to wild-type *B. marisflavi* 151–25, the transcript levels of seven genes showed marked fold increases in 151–25△3 (Additional file 12: Figure S7a). These genes were *orf666* (TetR family transcriptional regulator gene), *orf667* (cysteine ABC transporter substrate-binding protein gene), *orf668* (ABC transporter permease gene), *orf1240* (ArsR family transcriptional regulator gene), *orf1241* (copper-translocating P-type ATPase gene), *orf3892* (hypothetical protein gene) and *orf3894* (cation transporter gene). To confirm whether these genes were related toCd^2+^ resistance for *B. marisflavi* 151–25, three gene fragments also with *4802–4803* were overexpressed in *B. subtilis* and the Cd-MIC values for those recombinant strains were determined. As shown in Additional file 12: Figure S7b, the operon containing *orf4802* and *orf4803* allowed recombinant *B. subtilis* to exhibit greater Cd^2+^ resistance, while the other three fragments would not increase Cd^2+^ resistance of the recombinant *B. subtilis*. Our results suggested that the operon of 4*802–4803* plays a leading role for Cd^2+^ resistance of *B. marisflavi* 151–25, and that when plasmid p25 (containing the main Cd-efflux pump genes, *orf4802* and *orf4803*) was deleted, the transcript level of other efflux pump genes would be enhanced. The interaction between these genes remains to be further study.

### A gene cluster consisting of *orf4111*, *orf4112* and *orf4113* confers Cd^2+^ resistance for *B. vietamensis* 151–6

**Fig. 5:**
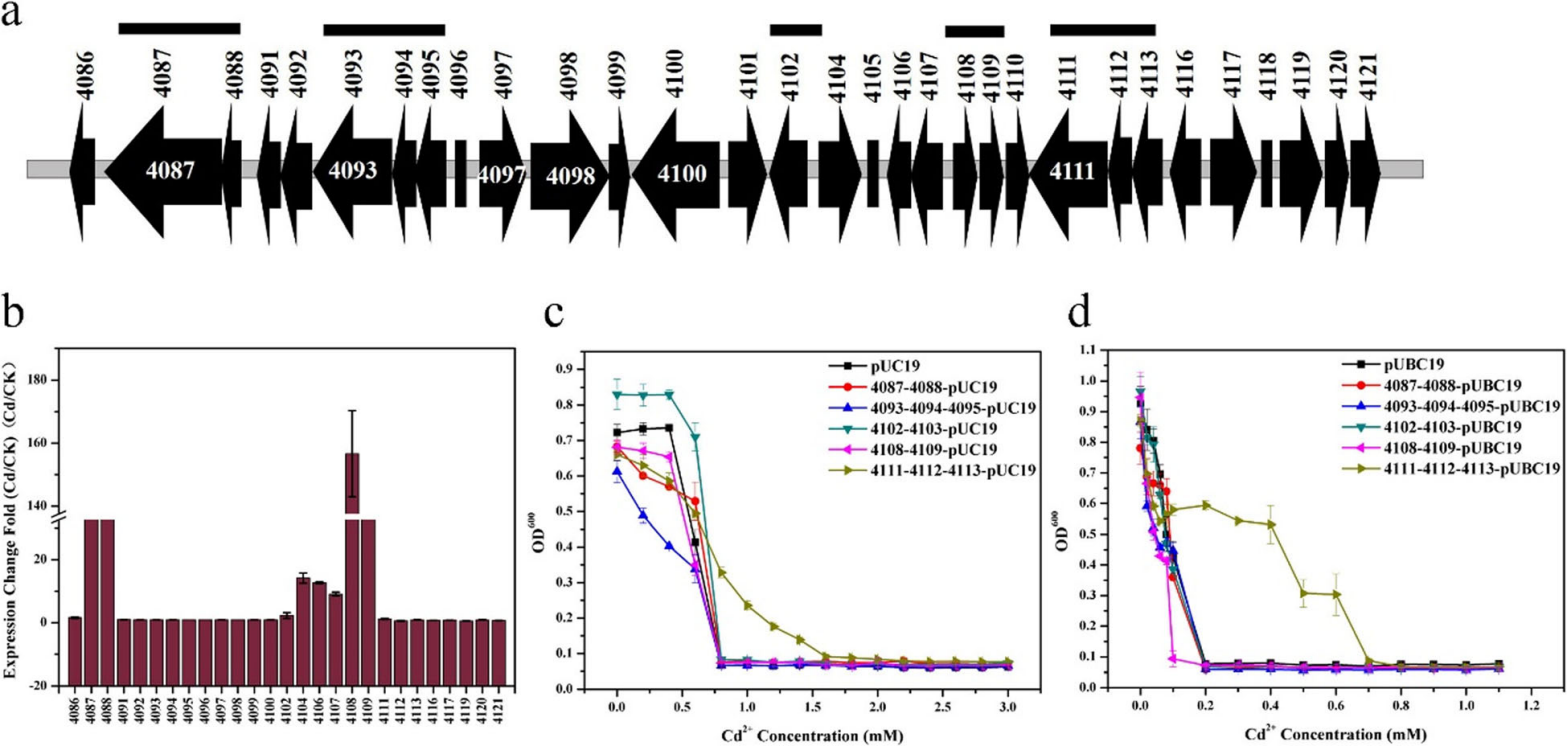
Genetic organization and transcription analysis of insert genes in fosmid clone B2 and examination of MIC-Cd for recombinant *E. coli* and *B. subtilis* containing the gene fragments of *B. vietamensis* 151–6. **a** Schematic representation of the insert fragments loci of fosmid clone B2. Thick gray line, *B. vietamensis* 151–6 chromosomal DNA; filled arrows and square, 31 open reading frames with corresponding gene size; black horizontal line, overexpressed gene fragments. **b** Transcription analysis of the insert genes in fosmid clone B2 in *B. vietamensis* 151–6 cultured with 0.1 mM Cd^2+^ (Cd) in comparison with a culture grown in the absence of Cd^2+^ (CK) using quantitative reverse transcription-polymerase chain reaction (qRT-PCR). Transcript levels of tested genes were normalized to the 16S rRNA gene. **c** Determination of MIC-Cd for recombinant *E. coli* containing the vectors pUC19 (negative control), 4087–4088-pUC19, 4093–4094-4095-pUC19, 4102–4103-pUC19, 4108–4109-pUC19 and 4111–4112-4113-pUC19 with varying concentrations of Cd^2+^ (0, 0.2, 0.4, 0.6, 0.8, 1.0, 1.2, 1.4, 1.6, 1.8, 2.0, 2.2, 2.4, 2.6, 2.8 and 3.0 mM). **d** Determination of MIC-Cd for recombinant *B. subtilis* containing the vectors pUBC19 (negative control), 4087–4088- pUBC19, 4093–4094-4095-pUBC19, 4102–4103-pUBC19, 4108–4109-pUBC19 and 4111–4112-4113-pUBC19 with varying concentrations of Cd^2+^ (0, 0.02, 0.04, 0.06, 0.08, 0.1, 0.2, 0.3, 0.4, 0.5, 0.6, 0.7, 0.8, 0.9, 1.0 and 1.1 mM)

Compared to *B. marisflavi* 151–25, the analysis of the transcriptome of *B. vietamensis* 151–6 under Cd^2+^ induction demonstrated that a total of 1268 differentially expressed genes were identified (585 up-regulated genes and 683 down-regulated genes), significantly higher than that of *B. marisflavi* 151–25 (Additional file 13: Figure S8 and Additional file 5: Table S5). Moreover, 585 up-regulated genes in the presence in Cd^2+^ were located on the chromosome, and among the 683 down-regulated genes, only 16 were located on plasmid p6. These data verified that plasmid p6 does not influence the Cd^2+^ resistance of *B. vietamensis* 151–6. To further identify the key Cd^2+^ resistance genes from those differentially expressed genes, a fosmid library of *B. vietamensis* 151–6 genomic DNA was constructed in *E. coli*. And the clones were screened by the Cd^2+^ resistance to determine the genes that contribute to Cd^2+^ resistance. Among the total of 1204 clones, there were 25 and 3 clones were able to grown on LB agar plates with 1.2 mM and 1.5 mM Cd^2+^, respectively. In liquid LB medium with 1.2 mM Cd^2+^, there were 27 clones could be grown. Only 2 clones, B2 and C2, could be grown in liquid LB medium with 1.5 mM Cd^2+^. The MIC-Cd of B2 and C2 were determined. As shown in Additional file 14: Figure S9, B2 and C2 exhibit higher Cd^2+^ resistance than negative control strain EPI300-T1^R^. The fosmid DNAs isolated from B2 and C2 were sequenced. Sequence analyses of B2 revealed that it contained 32,263 bp insert fragment, which consisted 31 annotated genes (Table 2) (And the fosmid DNA isolated from C2 was failed by sequencing many times, so the clone was not analyzed). Moreover, the analysis of the transcriptome showed that insert fragment of B2 contained 8 up-regulated genes (*orf4108, orf4109, orf4088, orf4087, orf4090, orf4093, orf4106, orf4107*) and 3 down-regulated genes (*orf4086, orf4101, orf4120*). These results were confirmed through qRT-PCR and the transcription levels of other 16 genes in the insert fragment of B2 were also evaluated by qRT-PCR. The results presented that the transcripts of the *orf4108*, *orf4109*, *orf4088*, *orf4087*, *orf4104*, *orf4106* and *orf4107* were increased 156.64, 130.84, 102.58, 87.26, 14.19, 12.68 and 9.07-fold, respectively (Table 2 and Fig. 5b). Based on these results, four gene clusters (*4108–4109*, *4087–4088*, *4104*, *4106–4107*) containing their own promoter were overexpressed in *E. coli* and *B. subtilis* to examine their contributions to the Cd^2+^ resistance. Due to the fact that ATPase gene and oxidoreductase gene have been reported to be involved in Cd^2+^ resistance [32], three related gene clusters (*4093–4094-4095*, *4102–4103* and *4111–4112-4113*) containing their own promoter were also overexpressed in *E. coli* and *B. subtilis* (And the construction of the recombinant vectors containing *4104* and *4106–4107* were failed by many times, so the results were not showed). As shown in Fig. 5c and d, the gene cluster containing *orf4111* (copper-translocating P-type ATPase gene), *orf4112* (cadmium efflux system accessory protein gene, *cadC*) and *orf4113* (cadmium resistance protein gene, *cadD*) allowed recombinant *E. coli* and *B. subtilis* to exhibit higher Cd^2+^ resistance than the negative control strain. Especially, the *B. subtilis* cells containing the recombinant plasmid 4111–4112-4113-pUBC19 demonstrated the potential to tolerate and grow in the presence of 6 times the concentration of Cd^2+^ compared to the control cells. Moreover, the recombinant strain exhibited similar Cd resistance with the strain which overexpressed the gene fragment *4802–4803* from *B. marisflavi* 151–25 (Fig. 4d). However, compared to the negative control cells, the other 4 recombinant *E. coli* and *B. subtilis* cells did not show great Cd^2+^ resistance. These results indicated that the gene cluster consisting of *orf4111*, *orf4112* and *orf4113* confers Cd^2+^ resistance for *B. vietamensis* 151–6.

**Table 2:** Gene annotation information, RNA sequencing and qRT-PCR analysis of insert fragment of B2

Gene	Location		NR annotation	RNA sequence Fold Change (Cd/CK)	qRT-PCR Fold change (Cd/CK)
	Start	End			
*orf4086*	3,723,301	3,723,687	four-helix bundle copper-binding protein	−1.95	1.59
*orf4087*	3,723,960	3,726,575	ATPase	47.79 ^b^	87.26 ^b^
*orf4088*	3,726,588	3,726,953	transcriptional regulator	105.62 ^a^	102.58 ^a^
*orf4090*	3,727,300	3,727,605	hypothetical protein	2.06	N
*orf4091*	3,727,630	3,728,040	hypothetical protein	/	0.96
*orf4092*	3,728,051	3,728,596	cysteine ABC transporter ATP-binding protein	/	0.89
*orf4093*	3,728,764	3,730,818	cadmium transporter	1.85	0.89
*orf4094*	3,730,891	3,731,259	transcriptional regulator	/	0.89
*orf4095*	3,731,357	3,731,866	hypothetical protein	/	0.89
*orf4096*	3,732,138	3,732,377	ferrous iron transport protein A	/	0.89
*orf4097*	3,732,378	3,733,106	small GTP-binding protein domain-containing protein	/	0.89
*orf4098*	3,733,084	3,734,481	ferrous iron transport protein B	/	0.89
*orf4099*	3,734,546	3,734,869	small multidrug resistance family-3 protein	/	0.89
*orf4100*	3,734,912	3,736,732	MULTISPECIES: hypothetical protein	/	0.89
*orf4101*	3,736,938	3,737,783	hypothetical protein	−2.71	N
*orf4102*	3,737,798	3,738,649	cation diffusion facilitator family transporter	/	2.29
*orf4104*	3,738,917	3,739,834	YIEGIA protein	/	14.19
*orf4105*	3,739,859	3,740,047	hypothetical protein	/	N
*orf4106*	3,740,119	3,740,538	disulfide bond formation protein B	13.10 ^b^	12.68 ^b^
*orf4107*	3,740,539	3,741,201	Protein-disulfide isomerase	12.17 ^b^	9.07
*orf4108*	3,741,422	3,741,952	thiol-disulfide oxidoreductase	372.96 ^a^	156.64 ^a^
*orf4109*	3,742,073	3,742,675	cytochrome C biogenesis protein CcdA	194.33 ^a^	130.84 ^a^
*orf4111*	3,743,680	3,745,806	copper-translocating P-type ATPase	/	1.20
*orf4112*	3,745,799	3,746,164	cadmium efflux system accessory protein CadC	/	0.59
*orf4113*	3,746,292	3,746,906	cadmium resistance protein CadD	/	0.89
*orf4116*	3,747,815	3,748,807	oxidoreductase	/	0.73
*orf4117*	3,749,310	3,750,713	hypothetical protein	/	0.77
*orf4118*	3,750,703	3,750,882	hypothetical protein	/	N
*orf4119*	3,750,879	3,752,096	alpha-hydroxy-acid oxidizing enzyme	/	0.57
*orf4120*	3,752,206	3,752,757	hypothetical protein	−2.12	0.87
*orf4121*	3,752,778	3,753,803	ion transporter	/	0.69

/represent the fold change of transcriptome is not significant; N represent qRT-PCR is not performed because the target genes are too small; ^a^represent the fold change of target gene > 100; ^b^ represent the fold change of target gene > 10

## Discussion

The present study showed that the gene cluster 4802–4803 located onA plasmid p25 and the gene cluster 4111–4112-4113 located on chromosome were involved in Cd^2+^ resistance for strain *B. marisflavi* 151–25 and *B. vietamensis* 151–6, respectively. Many microorganisms have been reported to use heavy-metal-transporting ATPases, such as the proteins CadA and ZntA, to overcome Cd^2+^ toxicity [19, 33]. Specifically, CadA from *S. aureus* has a length of 727 amino acids [19], and its amino acid sequence is highly similar to 4111 (63.31%) and 4802 (65.21%) (Additional file 15: Figure S10). Alignment with CadA from *S. aureus* showed that the protein sequences of *orf4111* from *B. vietamensis* 151–6 and *orf4802* from *B. marisflavi* 151–25 all included a conserved (Cys-Pro-Cys) tripeptide for Cd^2+^, Pb^2+^ or Zn^2+^ binding [34]. CadC from *S. aureus* including 122 amino acids was related to the divalent cation ATPase [27]. Expression of CadA is regulated by CadC, which is a homodimeric repressor that dissociates from the *cad* operator/promoter upon Cd^2+^ binding [19]. CadC is a member of the ArsR/SmtB family of metalloregulatory proteins [29, 30]. Its crystal structure was resolved in 2005, and showed that two regulatory metal-binding sites for the inducer Cd^2+^ are formed by Cys-7 and Cys-11 from the N terminus of one monomer and Cys-58 and Cys-60 of the other monomer [34]. Alignment with CadC from *S. aureus* showed that the protein sequence of *orf4112* from *B. vietamensis* 151–6 and of *orf4803* from *B. marisflavi* 151–25 contained these four Cys residues (Additional file 16: Figure S11). Moreover, the similarity between CadC and Orf4803 or Orf4112 are 86.89 and 82.64%, respectively. These analysis indicated that the *orf4111*, *orf4112* from *B. vietamensis* 151–6 and *orf4802*, *orf4803* from *B. marisflavi* 151–25 all encoded the proteins CadA and CadC, respectively. The protein CadA existed as the efflux transporter and was vital to the cadmium resistance. And the protein CadC was a transcription factor to regulate the expression of CadA. However, the expression mechanism of the two proteins in *B. vietamensis* 151–6 was different to the *B. marisflavi* 151–25. In *B. marisflavi* 151–25, the two genes (*orf4802* and *orf4803*) were induced by the cadmium. While in *B. vietamensis* 151–6, the expression of CadC (Orf4112) was repressed by the cadmium, as the qRT-PCR Fold change (Cd/CK) was only 0.56 (Table 2). As a result, few CadC was expressed in the cell and the expression of CadA (Orf4111) was activated by the cadmium and endowed the cadmium function of the organism, which qRT-PCR Fold change (Cd/CK) of *orf4111* was 1.2 (Table 2). Those analysis indicated that the cadmium resistance mechanism of *B. marisflavi* 151–25 was the same as *cad* system in *S. aureus*. The hypothetical expression model showed in Additional file 17: Figure S12. Additionally, we did not know the detailed mechanism about the expression of *orf4111–41,112* in *B. vietamensis* 151–6. In the future study, we will clone the promoter and study the regulation of the *cad* system.

In addition, the result of the transcriptome of *B. marisflavi* 151–25 under Cd^2+^ induction showed that *orf4608* (arsenical efflux pump membrane protein gene, *arsB*) and *orf4779* (arsenic resistance protein gene) were all induced by Cd^2+^. However, overexpression of two genes in *E. coli* did not improve Cd^2+^ resistance of recombinant *E. coli*. Maybe these genes can confer arsenic resistance for *B. marisflavi* 151–25. Presently, it has been reported that many *Bacillus* sp. strains (such as *B. subtilis*, *B. safensis* and *B. cereus*) have ability of resisting arsenic [35]. And the work of arsenic resistance for *B. marisflavi* 151–25 will be carried out in future study.

## Conclusions

In our study, we isolated two different Cd^2+^ resistance strains *B. vietamensis* 151–6 and *B. marisflavi* 151–25 isolated from Cd-contaminated soil, which could grow in the presence of Cd^2+^ at two distinct concentrations up to 0.3 mM and 0.8 mM, respectively. Further study with whole genome sequencing, transcriptome sequencing and heterologous expression revealed a gene cluster that played a vital role in Cd^2+^ resistance of the two strains. Orf4802 (ATPase transporter) and Orf4803 (transcriptional regulator) on plasmid p25, were found to be major contributors to Cd^2+^ resistance in *B. marisflavi* 151–25. And Orf4111 (ATPase transporter), Orf4112 (cadmium efflux system accessory protein) and Orf4113 (cadmium resistance protein) on the chromosome, were proved to be critical for Cd^2+^ resistance of *B.vietamensis* 151–6. In the end, we explored the cadmium resistance mechanism for *B. vietamensis* 151–6 and *B. marisflavi* 151–25. And we identified that the Cd^2+^ resistance mechanism of *B. marisflavi* 151–25 was the same as *cad* system in *S. aureus*. Although *Bacillus vietamensis* 151–6 also had the similar gene cluster to *B. marisflavi* 151–25 and *S. aureus*, its transcriptional regulatory mechanism of cadmium resistance was not same. This study could expand our understanding of the biological effects of cadmium.

## Methods

### Bacterial strains, plasmids, and growth conditions

The bacterial strains and plasmids used in this study are listed in Additional file 1: Table S1. *B. subtilis* WB600 (BS), *B. amyloliquefaciens* (BA) and *B. licheniformis* WX-02 (BL) were stored in our lab. *E. coli* TOP10 (TIANGEN Biotech, Beijing, China) was used as the cloning host. *B. vietamensis* 151–6 and *B. marisflavi* 151–25 were isolated from Cd-contaminated soil in Hunan Province (27°46′N, 112°52′E), China. These strains were all grown in Luria–Bertani (LB) medium. CdCl_2_·H_2_O was used as the source of Cd. Ampicillin (100 μg mL^− 1^), kanamycin (10 μg mL^− 1^), or tetracycline (5 μg mL^− 1^) was added as necessary.

### Isolation of cd-resistant bacteria

Cd-contaminated soil was collected from a former industrial site in Hunan Province (27°46′N, 112°52′E) and analyzed for Cd content through acid digestion followed by the use of a 7700 × inductively coupled plasma mass spectrometer (Agilent Technologies, Tokyo, Japan). To isolate Cd-resistant and bio-safe bacteria, aerobic *Bacillus* sp. were isolated from the soil by plating on LB agar plates containing progressively higher concentrations of cadmium chloride (0, 0.5, 1 and 2 mM). Then, in order to isolate aerobic *Bacillus* sp., the bacterial enrichment cultures were heat-shocked at 80 °C for 20 min. Bacterial genomic DNA was isolated using the TIANamp Bacteria DNA kit (TIANGEN Biotech). The 16S rRNA gene was amplified from the extracted DNA using the universal primers 16S rRNA-F/16S rRNA-R (Additional file 2: Table S2) and the amplification products were cloned in the pGM-T (TIANGEN Biotech) vector using competent *E. coli* TOP10 cells (TIANGEN Biotech). Sequencing was carried out using T7 and SP6 primers and compared to the GenBank database using the NCBI BLAST program.

### Evaluation of cadmium resistance and growth curve

To evaluate growth in a liquid medium of isolated bacteria, the minimum inhibitory concentration (MIC) of Cd^2+^ (MIC-Cd) was determined. LB medium (800 μL) with different concentrations of Cd^2+^ was dispensed into 96-well (12 × 8) microtiter plates (96 × 2-mL wells) with a multi-channel micropipette (rows A to H: 0 mM, 0.02 mM, 0.04 mM, 0.06 mM, 0.08 mM, 0.1 mM, 0.2 mM,0.3 mM, 0.4 mM, 0.5 mM, 0.6 mM, 0.7 mM, 0.8 mM, 0.9 mM, 1.0 mM and 1.1 mM). Single colonies of the test strains were inoculated into 3 mL of LB medium and cultured overnight. The test culture (15 μL) was then inoculated into each well of the prepared 96-well plate. After 24 h at 37 °C and 750 rpm in an incubator (Heidolph, Viertrieb, Germany), 200 μL of the cell suspension was transferred to a 96-well plate and the turbidity at OD_600_ was measured.

To determine the Cd^2+^ tolerance of isolated bacteria, growth curves at different concentrations (0 mM, 0.1 mM, 0.3 mM and 0.5 mM) of Cd^2+^ were analyzed. For the growth assay, single colonies of the test strains were cultured overnight and then diluted 1:100 into 100-well plates containing 200 μL of LB and various concentrations of Cd^2+^ in quintuplicate. The growth curve was measured at 1-h intervals using a Bioscreen C automatic growth curve analyzer (Bioscreen, Helsinki, Finland).

### Genome sequencing and analysis

Bacterial genomic DNA was extracted using the sodium dodecyl sulfate (SDS) method [36]. A total of 5 μg DNA was used to generate each library, and this DNA was sheared using Covaris g-Tubes to generate sheared fragments > 10 kb in length. The sheared DNA fragments were then prepared using the SMRT bell template preparation kit (Pacific Biosciences, Menlo Park, CA, USA) according to the manufacturer’s instructions. Whole-genome sequencing was performed on the Pacbio RSII platform. All high-quality paired reads were assembled using SOAPdenovo (http://soap.genomics.org.cn/soapdenovo.html) onto a number of scaffolds [37]. Then, the filtered reads were transferred for the next step of gap closing. Transfer RNA (tRNA) genes were predicted with tRNAscan-SE [38]. Ribosomal RNA (rRNA) genes were analyzed using rRNAmmer [39]. Coding genes were identified with the GeneMarkS program [40]. The predicted coding genes were annotated based on the non-redundant protein database (NR) of the National Center for Biotechnology Information [41] and Gene Ontology (GO) [42].

### RNA sequencing and transcriptome analysis

An overnight culture was diluted 1:100 in LB medium in the presence (0.1 mM) and absence of Cd^2+^, and these cultures were grown at 37 °C and 200 rpm to the exponential phase. Total RNA from both groups (each group has three replicates) were extracted using a TRIzol kit (TIANGEN Biotech) according to the manufacturer’s instructions. A total of 1 μg RNA per sample was used as input material for RNA sample preparation. Sequence libraries were generated using the Illumina® TruSeq® Stranded Total RNA Sample Preparation kit (NEB, Ipswich, MA, USA). TruSeq Stranded Total RNA was prepared with the Ribo-Zero™ Bacteria Kit (Epicenter, Madison, WI, USA). The clustering of index-coded samples was performed on a cBot Cluster Generation System using the TruSeq HiSeq 4000 PE 150 Cluster Kit (Illumina, San Diego, CA, USA) according to the manufacturer’s instructions. After cluster generation, the prepared libraries were sequenced on the Illumina HiSeq 4000 platform by Allwegene Technology (Beijing, China) and 150-bp paired-end reads were generated. Bowtie2 was used to map mRNA reads to the genome, and HTSeq v 0.5.4 p3 was used to count the number of reads mapped to each gene. Then, the fragments per kilobase of exon model per million mapped reads (FPKM) of each gene was calculated based on the length of, and read count mapped to, the gene [43].

### Elimination of plasmids

Plasmid elimination was performed using SDS, as previously described [44], with some modifications. Specifically, *B. vietamensis* 151–6 and *B. marisflavi* 151–25 were treated with 0.0005 and 0.002% SDS, respectively. Single colonies were selected on LB agar plates to screen for strains that had eliminated the p6 plasmid or the p25 plasmid. Primers 4108CDS-F/4108CDS-R and 4109CDS-F/4109CDS-R (Additional file 2: Table S2) were used to identify gene located on the chromosome of *B. vietamensis* 151–6. The primer pairs 4963CDS-F/4963-CDS-R, 4967CDS-F/4967CDS-R, 4982CDS-F/4982CDS-R, 4983CDS-F/4983CDS-R, 5014CDS-F/5014CDS-R and 5018CDS-F/5018CDS-R (Additional file 2: Table S2) were employed to identify the genes in the plasmid p6 (Additional file 9: Figure S4) For *B. marisflavi* 151–25, the primers 4163CDS-F and 4163CDS-R (Additional file 2: Table S2) were used to identify genes located on the chromosome, while the primer pairs 4779CDS-F/4779-CDS-R, 4780CDS-F/4780CDS-R and 4803CDS-F/4803CDS-R (Additional file 2: Table S2) were used to detect the p25 plasmid (Additional file 10: Figure S5). Meanwhile, the primers for quantitative reverse transcription-polymerase chain reaction (qRT-PCR) (Additional file 2: Table S2) located on plasmid p25 were also used to detect the plasmid (Additional file 10: Figure S5).

### RT-PCR and qRT-PCR

Total RNA of *B. vietamensis* 151–6 and *B. marisflavi* 151–25 were isolated as described above under RNA sequencing. cDNA samples were prepared using TransScript One-Step gDNA Removal and cDNA Synthesis SuperMix (TransGen Biotech, Beijing, China) with random primers according to the manufacturer’s instructions. qRT-PCR was performed using the ChamQ Universal SYBR qPCR Master Mix (Vazyme Biotech, Beijing, China) according to the manufacturer’s instructions. 16S rRNA, amplified with the primers 16S-F/16S-R, was used as an internal control. All reactions were performed in biological triplicate, and the normalized fold changes of the relative expression ratios were quantified using the 2^-△△CT^ method [45].

### Construction and screening of an *B. vietamensis* 151–6 genomic fosmid library

High-molecular-weight genomic DNA from *B. vietamensis* 151–6 was extracted using the bacterial genomic DNA extraction kit (BioTeke Biotech, Beijing, China) according to the manufacture’s instructions. And a fosmid library was constructed with the CopyControl Fosmid Library Production Kit (vector pCC1FOS; Epicentre, Madison, WI, USA) according to the manufacture’s instructions. A total of 1204 clones were used to construct the library in 13 96-well plates. To screen for clones with Cd^2+^ resistance, overnight cultures of the 1204 clones were inoculated into LB liquid medium with 1.0 mM and 1.2 mM Cd^2+^, respectively. Meanwhile, these clones were spotted onto LB agar plates with 1.2 mM and 1.5 mM Cd^2+^. Fosmid DNA was isolated from the positive fosmid clones by Axyprep Plasmid Miniprep Kit (Axygen, USA) and sequenced by pCC1 sequencing primers pCC1-F/ pCC1-R (Additional file 2: Table S2). The results were aligned with the genome sequence of *B. vietamensis* 151–6 to confirm the sequence of the inserted fragments.

### Construction of plasmids for gene fragments heterologous expression

To verify that Cd^2+^ resistance is conferred by genes, recombinant pUC19 and pUBC19 plasmids were constructed. The 3444-bp *Sac* I-*Xba* I DNA fragment amplified from *B. vietamensis* 151–6 genome with the primers 4111–4112-4113-F/4111–4112-4113-R (Additional file 2: Table S2), containing copper-translocating P-type ATPase gene (*orf4111*), cadmium efflux system accessory gene *cadC* (*orf4112*), cadmium resistance gene *cadD* (*orf4113*) and 217-bp upstream of *orf4113*, was cloned into *E. coli* pUC19 vector and *E. coli*-*B. subtilis* shuttle vector pUBC19, respectively. The gene fragments *4087–4088*, *4093–4094-4095*, *4102–4103* and *4108–4109* amplified from *B. vietamensis* 151–6 genome with corresponding primers (Additional file 2: Table S2) were also cloned into pUC19 and pUBC19. For *B. marisflavi* 151–25, the gene fragments *4774–4775*, *4776–4777*, *4779–4780*, *4781–4782* and *4802–4803* amplified from plasmid p25 of *B. marisflavi* 151–25 with the corresponding primers (Additional file 2: Table S2) were cloned into pUC19 and pUBC19. The ligation mixture was transformed into *E. coli* TOP10, and the correct plasmids were identified through colony PCR with corresponding sequencing primers (Additional file 2: Table S2). After confirmation via sequencing, recombinant pUBC19 plasmids were extracted from *E. coli* TOP10 and transformed into *B. subtilis*, as described previously [46]. Transformants were harvested by screening the clones on LB agar containing 10 μg/mL kanamycin, and the correct plasmids were verified through colony PCR with the pUBC19 sequencing primers CX-F/CX-R (Additional file 2: Table S2).

### MIC-Cd and growth curve determination

MIC-Cd and the growth curve of plasmid-eliminated strains and wild strains of *B. vietamensis* 151–6 and *B. marisflavi* 151–25 were determined as described above in 2.3. MIC-Cd values for the recombinant strains of *E. coli* TOP10 and *B. subtilis* were also evaluated using 96-well microtiter plates, as described in 2.3.

## Supplementary information

**Additional file 1: Table S1.** Bacterial strains and plasmids used in this study.

**Additional file 2: Table S2.** Primers used in this study.

**Additional file 3: Table S3.** Cd-MIC and 16S rDNA identification for isolating strains.

**Additional file 4: Table S4.** Up-regulating genes and down-regulating genes in the presence of Cd^2+^ by RNA sequencing for 151–25.

**Additional file 5: Table S5.** Partial up-regulating genes (fold change> 10) and down-regulating genes (fold change> 20) in the presence of Cd^2+^ by RNA sequencing for 151–6.

**Additional file 6: Figure S1.** Growth curve of *Bacillus sp.* strains (151–6, 151–25, *B. subtilis* WB600 (BS), *B. amyloliquefaciens* (BA) and *B. licheniformis* WX-02 (BL)) at different concentrations of cadmium.

**Additional file 7: Figure S2.** Genome map and plasmid map of 151–6.

**Additional file 8: Figure S3.** Genome map and plasmid map of 151–25.

**Additional file 9: Figure S4.** Verification of the elimination of the plasmid p6 for 151–6.

**Additional file 10: Figure S5.** Verification of the elimination of the plasmid p25 for 151–25.

**Additional file 11: Figure S6.** Volcano map of differentially expressed genes of 151–25.

**Additional file 12: Figure S7.** Transcription analysis of up-regulated genes based on RNA sequencing and examination of Cd-MIC for recombinant *B. subtilis* containing the operon of 151–25.

**Additional file 13: Figure S8.** Volcano map of differentially expressed genes of 151–6.

**Additional file 14: Figure S9.** Evaluation of Cd-MIC of B2 and C2. B2 and C2 were screened clones in *E. coli* fosmid library for 151–6. EPI300-T1R was negative control *E. coli* strain.

**Additional file 15: Figure S10.** Alignment of the putative *cadA* amino acid sequence (4111, 4802) from 151 to 6 and 151–25 versus the CadA from *S. aureus*.

**Additional file 16: Figure S11.** Alignment of the putative *cadc* amino acid sequence (4112, 4803) from 151 to 6 and 151–25 versus the CadC from *S. aureus*.

**Additional file 17: Figure S12.** The hypothetical *cad* system model of *B. marisflavi* 151–25

## Data Availability

The *B. vietamensis* 151–6 chromosome and plasmid p6, also the chromosomal and p25 plasmid sequences of *B. marisflavi* 151–25 have been deposited in GenBank of NCBI. The BioProject IDs of *B. vietamensis* 151–6 and *B. marisflavi* 151–25 in NCBI were PRJNA545803 and PRJNA490129, respectively. If you have any problems accessing the data, you can ask the corresponding author for help.

## Abbreviations

Cd: Cadmium
MIC: Minimum inhibitory concentration
MIC-Cd: Minimum inhibitory concentration of Cd^2+^
