# Supplementary material for: An operon consisting of a P-type ATPase gene and a transcriptional regulator gene responsible for cadmium resistances in Bacillus vietamensis 151–6 and Bacillus marisflavi 151–25
Source: BMC Microbiol. 2020 Jan 21;20:18. doi: 10.1186/s12866-020-1705-2 (PMC6975044; doi:10.1186/s12866-020-1705-2)
Supplement: Supplementary file 1 — Additional file 1: Table S1. Bacterial strains and plasmids used in this study. [file 12866_2020_1705_MOESM1_ESM.docx]

**Table S1.** Bacterial strains and plasmids used in this study.

| Strains | Description | Reference or Source |
| --- | --- | --- |
| *E. coli* Top 10 | Clone strain | Our lab |
| *B. subtilis* WB600 |  | Our lab |
| *B. amyloliquefaciens* |  | Our lab |
| *B. licheniformis* WX-02 |  | Our lab |
| *B. vietamensis* |  | This study |
| 151-6 | Wild type | This study |
| 151-6△5 and 151-6△7 | The p6 plasmid was deleted | This study |
| *B. marisflavi* |  | This study |
| 151-25 | Wild type | This study |
| 151-25△3 and 151-25△29 | The p25 plasmid was deleted | This study |
| Plasmids |  |  |
| pUC19 | *E. coli* clone vector | Our lab |
| 4087-4088- pUC19 | 151-6 chromosomal fragment containing *orf4087*, *orf4088* and 230-bp upstream of *orf4088* were inserted into pUC19 |  |
| 4093-4094-4095- pUC19 | 151-6 chromosomal fragment containing *orf4093*, *orf4094*, *orf4095* and 271-bp upstream of *orf4095* were inserted into pUC19 |  |
| 4102-4103- pUC19 | 151-6 chromosomal fragment containing *orf4102*, *orf4103* and 262-bp upstream of *orf4103* were inserted into pUC19 |  |
| 4108-4109- pUC19 | 151-6 chromosomal fragment containing *orf4108*, *orf4109* and 220-bp upstream of *orf4108* were inserted into pUC19 |  |
| 4111-4112-4113- pUC19 | 151-6 chromosomal fragment containing *orf4111*, *orf4112*, *orf4113* and 217-bp upstream of *orf4113* were inserted into pUC19 | This study |
| 4774-4775- pUC19 | 151-25 p25 plasmid frsgement containing *orf4774* and *orf4775* were inserted into pUC19 | This study |
| 4776-4777- pUC19 | 151-25 p25 plasmid frsgement containing *orf4776*, *orf4777* and 427-bp upstream of *orf4777* were inserted into pUC19 | This study |
| 4779-4780- pUC19 | 151-25 p25 plasmid frsgement containing *orf4779* and *orf4780* were inserted into pUC19 | This study |
| 4781-4782- pUC19 | 151-25 p25 plasmid frsgement containing *orf4781*, *orf4782* and 309-bp upstream of *orf4782* were inserted into pUC19 | This study |
| 4802-4803-pUC19 | 151-25 p25 plasmid frsgement containing *orf4802*, *orf4803* and 878-bp upstream of *orf4802* were inserted into pUC19 | This study |
| pUBC19 | *E. coli*-*B. subtilis* shuttle vector | Our lab |
| 4087-4088- pUBC19 | 151-6 chromosomal fragment containing *orf4087*, *orf4088* and 230-bp upstream of *orf4088* were inserted into pUBC19 |  |
| 4093-4094-4095- pUBC19 | 151-6 chromosomal fragment containing *orf4093*, *orf4094*, *orf4095* and 271-bp upstream of *orf4095* were inserted into pUBC19 |  |
| 4102-4103- pUBC19 | 151-6 chromosomal fragment containing *orf4102*, *orf4103* and 262-bp upstream of *orf4103* were inserted into pUBC19 |  |
| 4108-4109- pUBC19 | 151-6 chromosomal fragment containing *orf4108*, *orf4109* and 220-bp upstream of *orf4108* were inserted into pUBC19 |  |
| 4111-4112-4113- pUBC19 | 151-6 chromosomal fragment containing *orf4111*, *orf4112*, *orf4113* and 217-bp upstream of *orf4113* were inserted into pUBC19 | This study |
| 4774-4775- pUBC19 | 151-25 p25 plasmid frsgement containing *orf4774* and *orf4775* were inserted into pUBC19 | This study |
| 4776-4777- pUBC19 | 151-25 p25 plasmid frsgement containing *orf4776*, *orf4777* and 427-bp upstream of *orf4777* were inserted into pUBC19 | This study |
| 4779-4780- pUBC19 | 151-25 p25 plasmid frsgement containing *orf4779* and *orf4780* were inserted into pUBC19 | This study |
| 4781-4782- pUBC19 | 151-25 p25 plasmid frsgement containing *orf4781*, *orf4782* and 309-bp upstream of *orf4782* were inserted into pUBC19 | This study |
| 4802-4803-pUBC19 | 151-25 p25 plasmid frsgement containing *orf4802*, *orf4803* and 878-bp upstream of *orf4802* were inserted into pUBC19 | This study |
| 666-667-668-pUBC19 | 151-25 chromosome frsgement containing *orf666*, *orf667*, *orf668* and 268-bp upstream of *orf666* were inserted into pUBC19 | This study |
| 1240-1241-pUBC19 | 151-25 chromosome frsgement containing *orf1240*, *orf1241* and 260-bp upstream of *orf1240* were inserted into pUBC19 | This study |
| 3892-3894-pUBC19 | 151-25 chromosome frsgement containing *orf3892*, *orf3893*, *orf3894* and 303-bp upstream of *orf3894* were inserted into pUBC19 | This study |
