# Supplementary material for: An operon consisting of a P-type ATPase gene and a transcriptional regulator gene responsible for cadmium resistances in Bacillus vietamensis 151–6 and Bacillus marisflavi 151–25
Source: BMC Microbiol. 2020 Jan 21;20:18. doi: 10.1186/s12866-020-1705-2 (PMC6975044; doi:10.1186/s12866-020-1705-2)
Supplement: Supplementary file 2 — Additional file 2: Table S2. Primers used in this study. [file 12866_2020_1705_MOESM2_ESM.docx]

**Table S2.** Primers used in this study.

| Primer | Sequence (5’-3’)^a^ |
| --- | --- |
| Verification of the elimination of the plasmid p25 | |
| 4163CDS-F | TTGACAGAAGGGGAAGCGGTTG |
| 4163CDS-R | TTAGAATGGAGAAGAAAAGATA |
| 4779CDS-F | ATGGAGTTTACCACTAGCTATG |
| 4779CDS-R | TTAGTTGGTTGTTTTACGCATG |
| 4780CDS-F | GTGAACGGGCAAAGGAAGTACA |
| 4780CDS-R | TTATCCACAAGTGCCGGTTGGA |
| 4803CDS-F | GTGATTAAGAAAGATACTTGTG |
| 4803CDS-R | CTAGACATTGATCTTCACCTCT |
| Verification of the elimination of the plasmid p6 | |
| 4108CDS-F | ATGAAAAAAAGAAAGAGAAGATTGG |
| 4108CDS-R | TTATTGTTCTGGCTTGATCA |
| 4109CDS-F | ATGTCCATAGATGAATTAAATAACC |
| 4109CDS-R | CTAGAATCCCGTAAAACCAC |
| 4963CDS-F | GTGTTATCCGAAAACAAAAA |
| 4963CDS-R | TTATTCGAATACCTCCTCTG |
| 4967 CDS-F | ATGAAACATAAACCAATCGC |
| 4967 CDS-R | CTACTCATTAGGTCGAAACG |
| 4982 CDS-F | ATGAGCGTTCGGTTTCAATT |
| 4982 CDS-R | TCATTGCAACTCCTCTCTAA |
| 4983 CDS-F | ATGAATGAGCGATCGAAAGA |
| 4983 CDS-R | TCAAATTGAAACCGAACGCT |
| 5014 CDS-F | ATGTTCAGTGGTCCCGGGGTGAT |
| 5014 CDS-R | TTAGAAATCGAAACTCAATTGCT |
| 5018 CDS-F | GTGAGCCAAGTACCTTACAT |
| 5018 CDS-R | TCAGGCACTTCTACGTTTAG |
| 16S rRNA gene amplifying primers | |
| 16S rRNA-F | AGAGTTTGATCCTGGCTCAG |
| 16S rRNA-R | GGTTACCTTGTTACGACTT |
| pUC19 sequencing primers | |
| M13-F | CGCCAGGGTTTTCCCAGTCACGAC |
| M13-R | AGCGGATAACAATTTCACACAGGA |
| pUBC19 sequencing primers | |
| CX-F | GGTACAAGAAAAACGAGGAAAGATGC |
| CX-R | TCGTATGTTGTGTGGAATTGTGAGC |
| pCC1 sequencing primers | |
| pCC1-F | GGATGTGCTGCAAGGCGATTAAGTTGG |
| pCC1-R | CTCGTATGTTGTGTGGAATTGTGAGC |
| Operon of *B. marisflavi* 151-25 | |
| 4774-4775-F | ata**GAGCTC**ACGAACACAGCTCGATGACT |
| 4774-4775-R | ata**TCTAGA**CTACAGATCTTTAAAAGTCC |
| 4776-4777-F | ata**GAGCTC**TACTATTCTCTTCCTAATAGAA |
| 4776-4777-R | ata**TCTAGA**TCACTTGCTCCTGCCCTTCA |
| 4779-4780-F | ata**GGTACC**TTATCCACAAGTGCCGGTTGGAT |
| 4779-4780-R | ata**TCTAGA**TTAGTTGGTTGTTTTACGCATGA |
| 4781+4782-F | ata**GAGCTC**TGCCAAGAGACAAAAGACCA |
| 4781+4782-R | ata**TCTAGA**TTACTCCACCTTTCCCTTTG |
| 4082-4803-F | ata**GGTACC**TTTATATCAGTAGACTGTACTCT |
| 4082-4803-R | ata**TCTAGA**CTATTCTTTGATTTTCAATAAG |
| 666-667-668-F | ata**GGTACC**TTCAAAGTAAGCCGGATCCT |
| 666-667-668-R | ata**TCTAGA**TTATACATAGATTGATAGTCGC |
| 1240-1241-F | ata**GGTACC**ACTGCCGCCCTTCCTGATTT |
| 1240-1241-R | ata**TCTAGA**TCAACCCTGTTTCTTTTCCC |
| 3892-3894-F | ata**GGTACC**CTGAGGATCAGACGATCACG |
| 3892-3894-R | ata**TCTAGA**TTACCTTTGCTCTGAAGCTT |
| Operon of *B. vietamensis* 151-6 | |
| 4087-4088-F1 | ata**GGATCC**GCTTCAGAAGTTGGAACAAA |
| 4087-4088-R1 | ata**GTCGAC**CTATTTTACTTTTAAAAGGC |
| 4087-4088-F2 | ata**CTCGAG**GCTTCAGAAGTTGGAACAAA |
| 4087-4088-R2 | ata**TCTAGA**CTATTTTACTTTTAAAAGGC |
| 4093-4094-4095-F | ata**GAGCTC**TTGTTTTACCACCCTTCACATAT |
| 4093-4094-4095-R | ata**TCTAGA**TTATATAGCTTTTTGTTTTTCCCCC |
| 4102-4103-F | ata**GAGCTC**GTAATGATTTGAATAAGATAACC |
| 4102-4103-R | ata**TCTAGA**CTAGTTTTCTTTTATATGAATTTCT |
| 4108-4109-F | ata**GAGCT**CAAAAGTACACCTCTTCAAAATTATT |
| 4108-4109-R | ata**TCTAGA**CTAGAATCCCGTAAAACCACCAAAT |
| 4111-4112-4113-F | ata**GAGCTC**GCTTGAATGAGGGGATGGATAAA |
| 4111-4112-4113-R | ata**TCTAGA**TTATTCCTTTACTTTTAGTAACCGC |
| qRT-PCR (genes located on chromosomal of 151-6) | |
| 16S-F | ATCAGCGGCGGACGGGTGAG |
| 16S-R | GCGGGTCCATCTGTAAGTGA |
| 4086-F | CCCAAGGAGGAATGTAAGAT |
| 4086-R | ACCGAATGCACTCTTTCATC |
| 4087-F | GTACTGGAACGACCGAACAT |
| 4087-R | GGCAATCCATCCCATAAATC |
| 4088-F | GTGAAATTCCTGCATAGCTT |
| 4088-R | GCTGGGAAATGCTCGACTGG |
| 4091-F | TGCTTTCAATTGGGATTCTG |
| 4091-R | GGCGCTTACTTGATTGGTTT |
| 4092-F | TTGAAGCGGAAACAGATTGG |
| 4092-R | ATACGTCATTGGAGGGCAGA |
| 4093-F | TTCTGTTCGGGCTCAATGAA |
| 4093-R | TAAGCGAGCTTATGGGAACC |
| 4094-F | ACGAGTGATTGCGAAGACAT |
| 4094-R | TTCCGCAATCTGATTAACTG |
| 4095-F | CCGCATATTATTGGGAAGTA |
| 4095-R | GCAATGAATGGCAGCCTCCT |
| 4096-F | TTGAAGAAGGCAAAAGTATG |
| 4096-R | CGGTGGGATCGCCTAAAGGA |
| 4097-F | CTGGCCGGGTAAGACTGTAT |
| 4097-R | CAATATAATTCCGGGCGATT |
| 4098-F | CAACGCTCCTGATTGGCTTC |
| 4098-R | TGGGCAGATATCCGTAATTT |
| 4099-F | ATTTATCGTTGCCGGGCTGG |
| 4099-R | TCTGCAGAGTAGGGATGATC |
| 4100-F | TGTAGTCAGCCGGTTCGTCT |
| 4100-R | TTCTTACCGATCGCCACCAG |
| 4102-F | TTTGGGTGATAGTCTGTCGC |
| 4102-R | AATCGATCCGAACAGCAATA |
| 4104-F | TTATCGTAGCGCTCGTCACT |
| 4104-R | TTGCCTTTCGTGAATCTTTG |
| 4106-F | CGTTTCAATCATTGCGACAC |
| 4106-R | TCGCCACTCCCAGAATCAAC |
| 4107-F | GTGGTCGAGTTCGGTGATTT |
| 4107-R | TTCGCTTTCCCGGTATCAAT |
| 4108-F | CGAGCTATCATAGGGGAAGG |
| 4108-R | GCACCAAGTGCCCCAGAAGT |
| 4109-F | TAAAGAGTTGCTCCGCCAGA |
| 4109-R | CGTACCCAAATAACCAGATG |
| 4111-F | TGACCATCGCCATTATAGGA |
| 4111-R | TCAATAGACTGCCGAGCTTT |
| 4112-F | AGTGTTGCCCAAATGTTAAA |
| 4112-R | TTCGCGATATCACATACACA |
| 4113-F | ATTCCTCAACAATGGGCTAT |
| 4113-R | AAATGTTGCCTTCATCATCC |
| 4116-F | GTAGTGGCAACAGGACCTTT |
| 4116-R | CCTCCACCTACAACTAGAGC |
| 4117-F | TGAGAAATGGACCGAACAAA |
| 4117-R | GAAGTCTGCCATCGGGAAGC |
| 4119-F | CGCGACCTGAACGTGACTTT |
| 4119-R | AACGGGACATTCATGGATGC |
| 4120-F | CGCTATTTCGGCATGAGTCC |
| 4120-R | TGTCCCGATCATCTTGTTGG |
| 4121-F | TTTGTCACACTGGCAGCAGA |
| 4121-R | CTTCCCGTGCACGCTCATTC |
| qRT-PCR (genes located on plasmid p25) | |
| 16S-F | ATCAGCGGCGGACGGGTGAG |
| 16S-R | GCGGGTCCATCTGTAAGTGA |
| 4774-F | AGCTCGTTGGCATTAAGGTC |
| 4774-R | CGCGATCAACCAATCTGACA |
| 4775-F | CCGGGCTGTTTCGTTCTATC |
| 4775-R | CACATGCCATGCGATGAATA |
| 4776-F | CGGAATGAGGTAGAGAGCAC |
| 4776-R | CCCGTTGATGGATGACAGGT |
| 4777-F | CGCTTAAAGAAGATGAGAAA |
| 4777-R | TGCTCAAGCGATAATGGACA |
| 4779-F | CTAATCGCTTCACGCAAAGA |
| 4779-R | GGGCCACTAATCCAAATCCA |
| 4781-F | TGGCAGTAAGCTGGATGTCT |
| 4781-R | GGCCCTGTTCCTTGGAAATC |
| 4782-F | CGGACTGGCACTTAATGGAG |
| 4782-R | CCCGTTCTGTCCTGACATGA |
| 4802-F | GCCTATCGTGTTCAAGGATT |
| 4802-R | TCTTCAATGGTTGTCGTCCC |
| 4803-F | GTTGCCCAAATGTTAAAAGC |
| 4803-R | TCGCAGGTGATGAGAGGCAT |
| qRT-PCR (genes located on chromosomal of 151-25) | |
| 666-F | TGGTACTTCAAGAGCAAGGC |
| 666-R | GAGGCTGATACTGCATCCTG |
| 667-F | CGCCTTGACGTGAAAGTGAA |
| 667-R | TGCCATCTGCTTCTTACGTT |
| 668-F | CCGTCCGGTAAGATGGTTGG |
| 668-R | GCGATGTAGGCAGCCGTCAG |
| 1240-F | ATGCCATCGATGAAGAAACC |
| 1240-R | GTGAAAGCTTCTCGGCGATT |
| 1241-F | TCAGCGGATATCCCACGTTC |
| 1241-R | AAGCGTACCTTCCTTCCATT |
| 2329-F | TACGCAAAGAAGGAATGAAT |
| 2329-R | TTTACGCAGCGAAACTCCTC |
| 2552-F | AAATGGAATCGAGGCGCTAT |
| 2552-R | TTGGCAGAAAGGAAGATAAA |
| 2553-F | TGGATGGTGAGCCGCTTCTA |
| 2553-R | AGCCCATATGACGTTCTTCA |
| 2588-F | GAACGACGCCTCCACCTACA |
| 2588-R | TGAATACGACCGACGCAATC |
| 2757-F | ACAGGTGCTGAATGCGAAGG |
| 2757-R | GTGCGAACAAGGAAATCAGG |
| 3804-F | CGGATCGGGAATCAGATTGC |
| 3804-R | ATGCCCACCTGTTTCAGAAT |
| 3805-F | CTTCAAGCATCAAGGCAAAG |
| 3805-R | CTTCCGTTCATCCTTCAGGG |
| 3806-F | TGATCAAACGGGACCTGAAC |
| 3806-R | CGGCACTGACGGTGAAGACA |
| 3808-F | TGACGTCGCCCTCTCCAGTT |
| 3808-R | CATCGGCGATGGTGCTGTAA |
| 3892-F | TGTACATTATTGTGGCACAT |
| 3892-R | AAATACAATAAGCGCCAGGA |
| 3894-F | CGGCTACAAGCGGTTTGAGA |
| 3894-R | GCCGCTTGTAGCAACCTCAG |
| 4580-F | TGAAGGTATCGGAAATGAGC |
| 4580-R | GCGATCCCGTTAGAGGTCTT |
| 4581-F | TCAGTACGGAAGAAGGGTCA |
| 4581-R | TGTACCGGCCATCAATTCTC |
| 4582-F | GTGACCCTGCCAGACCCTTA |
| 4582-R | TCCGCTTCTTCCATCCCTTG |

^a^Restriction sites are indicated by blod characters.
