# Supplementary material for: An operon consisting of a P-type ATPase gene and a transcriptional regulator gene responsible for cadmium resistances in Bacillus vietamensis 151–6 and Bacillus marisflavi 151–25
Source: BMC Microbiol. 2020 Jan 21;20:18. doi: 10.1186/s12866-020-1705-2 (PMC6975044; doi:10.1186/s12866-020-1705-2)
Supplement: Supplementary file 3 — Additional file 3: Table S3. Cd-MIC and 16S rDNA identification for isolating strains. [file 12866_2020_1705_MOESM3_ESM.docx]

**Table S3.** Cd-MIC and 16S rDNA identification for isolating strains.

| Strains | Cd-MIC (mM) ^a^ | 16S rDNA identification |
| --- | --- | --- |
| 151-4 | 0.6 | *Uncutrured Bacillus* |
| 151-5 | 0.8 | *Bacillus aquimaris* |
| 151-6 | 0.4 | *Uncutrured Bacillus* |
| 151-7 | 0.6 | *Bacillus vietnamensis* |
| 151-8 | 0.4 | *Bacillus aquimaris* |
| 151-9 | 0.4 | *Uncutrured Bacillus* |
| 151-10 | 0.6 | *Bacillus sp.* |
| 151-11 | 0.8 | *Bacillus aquimaris* |
| 151-12 | 0.6 | *Bacillus vietnamensis* |
| 151-13 | 0.8 | *Bacillus sp.* |
| 151-15 | 0.6 | *Bacillus aquimaris* |
| 151-16 | 0.4 | *Bacillus aquimaris* |
| 151-17 | 0.4 | *Uncutrured Bacillus* |
| 151-18 | 0.4 | *Uncutrured Bacillus* |
| 151-19 | 0.4 | *Uncutrured Bacillus* |
| 151-20 | 0.4 | *Bacillus megaterium* |
| 151-21 | 0.6 | *Bacillus sp.* |
| 151-22 | 0.8 | *Bacillus aquimaris* |
| 151-23 | 0.4 | *Bacillus vietnamensis* |
| 151-24 | 0.6 | *Uncutrured Bacillus* |
| 151-25 | 1.0 | *Bacillus subtilis* |

^a^ The Cd-MIC was determined in LB liquid medium into 96-well (12×8) microtiter plates at varying concentrations of Cd^2+^ (0, 0.2, 0.4, 0.6, 0.8, 1.0, 1.2 and 1.4 mM).
