# Supplementary material for: An operon consisting of a P-type ATPase gene and a transcriptional regulator gene responsible for cadmium resistances in Bacillus vietamensis 151–6 and Bacillus marisflavi 151–25
Source: BMC Microbiol. 2020 Jan 21;20:18. doi: 10.1186/s12866-020-1705-2 (PMC6975044; doi:10.1186/s12866-020-1705-2)
Supplement: Supplementary file 4 — Additional file 4: Table S4. Up-regulating genes and down-regulating genes in the presence of Cd2+ by RNA sequencing for 151–25. [file 12866_2020_1705_MOESM4_ESM.docx]

**Table S4.** Up-regulating genes and down-regulating genes in the presence of Cd^2+^ by RNA sequencing for 151-25.

| Gene | Location | | Reads count | | Fold change (Cd/CK) | NR annotation |
| --- | --- | --- | --- | --- | --- | --- |
|  | Start | End | Cd | CK |  |  |
| Up-regulating genes | | | | | | |
| *orf4803^p^* | 51464 | 51832 | 500.24 | 6.90 | 72.53 | transcriptional regulator |
| *orf4802^p^* | 49342 | 51471 | 7460.73 | 116.89 | 63.83 | copper-translocating P-type ATPase |
| *orf4775 ^p^* | 26102 | 26236 | 70.79 | 1.65 | 42.98 | {NA} |
| *orf2588* | 2391209 | 2391628 | 42.29 | 1.10 | 38.54 | hypothetical protein |
| *orf4777 ^p^* | 28766 | 29110 | 45.73 | 1.75 | 26.10 | putative transcriptional regulator |
| *orf3805* | 3476608 | 3478050 | 69.69 | 2.83 | 24.65 | peptide ABC transporter permease |
| *orf3894* | 3552941 | 3553870 | 3165.92 | 129.46 | 24.45 | cation transporter |
| *orf4776 ^p^* | 26251 | 28752 | 3137.19 | 132.92 | 23.60 | ATPase |
| *orf4774 ^p^* | 25655 | 25972 | 566.50 | 26.48 | 21.40 | restriction endonuclease |
| *orf4580* | 4249832 | 4250452 | 1391.66 | 94.67 | 14.70 | DeoR faimly transcriptional regulator |
| *orf4581* | 4250468 | 4251811 | 6136.77 | 486.59 | 12.61 | PepSY-associated TM helix domain-containing protein |
| *orf4782 ^p^* | 31679 | 32254 | 899.96 | 76.98 | 11.69 | oxidoreductase |
| *orf4781 ^p^* | 31185 | 31682 | 619.15 | 54.15 | 11.43 | oxidoreductase |
| *orf4582* | 4251827 | 4252291 | 3097.60 | 276.43 | 11.21 | hypothetical protein |
| *orf2552* | 2356684 | 2357418 | 459.91 | 44.29 | 10.38 | MULTISPECIES: DNA-binding response regulator |
| *orf1241* | 1137314 | 1139347 | 5021.74 | 485.50 | 10.34 | copper-translocating P-type ATPase |
| *orf2553* | 2357415 | 2358818 | 1372.37 | 148.71 | 9.23 | two-component sensor histidine kinase |
| *orf1240* | 1136888 | 1137244 | 362.45 | 40.35 | 8.98 | ArsR family transcriptional regulator |
| *orf666* | 648625 | 649230 | 74.77 | 8.52 | 8.77 | TetR family transcriptional regulator |
| *orf3806* | 3478064 | 3479362 | 107.88 | 12.32 | 8.75 | peptide ABC transporter permease |
| *orf3892* | 3552375 | 3552578 | 26.56 | 3.42 | 7.76 | hypothetical protein |
| *orf2329* | 2148203 | 2148400 | 49.38 | 6.62 | 7.45 | hypothetical protein |
| *orf3808* | 3479596 | 3481386 | 269.99 | 46.51 | 5.80 | hypothetical protein |
| *orf0667* | 649283 | 650059 | 117.59 | 23.71 | 4.96 | cysteine ABC transporter substrate-binding protein |
| *orf2757* | 2529518 | 2530393 | 495.68 | 101.16 | 4.90 | iron ABC transporter |
| *orf4779 ^p^* | 29538 | 30608 | 1066.87 | 220.52 | 4.84 | arsenic resistance protein |
| *orf3804* | 3474740 | 3476446 | 283.54 | 66.72 | 4.25 | ABC transporter ATP-binding protein |
| *orf668* | 650074 | 650718 | 87.53 | 21.79 | 4.02 | MULTISPECIES: ABC transporter permease |
| *orf1647* | 1515491 | 1516297 | 541.26 | 144.59 | 3.74 | hypothetical protein |
| *orf3532* | 3220415 | 3221362 | 413.66 | 112.74 | 3.67 | cysteine synthase A |
| *orf3137* | 2851481 | 2852404 | 26.43 | 7.23 | 3.66 | MULTISPECIES: phosphate ABC transporter permease subunit PstC |
| *orf910* | 834192 | 835052 | 179.67 | 49.43 | 3.63 | hydrolase |
| *orf1648* | 1516278 | 1516976 | 124.96 | 35.81 | 3.49 | cysteine ABC transporter permease |
| *orf90* | 87689 | 88618 | 1630.79 | 496.04 | 3.29 | cysteine synthase A |
| *orf453* | 445229 | 445696 | 30.13 | 9.24 | 3.26 | {NA} |
| *orf1649* | 1517000 | 1517734 | 463.44 | 144.02 | 3.22 | ectoine/hydroxyectoine ABC transporter ATP-binding protein EhuA |
| *orf2758* | 2530390 | 2531649 | 500.12 | 155.88 | 3.21 | manganese ABC transporter permease |
| *orf669* | 650731 | 651471 | 133.80 | 47.56 | 2.81 | polar amino acid ABC transporter ATP-binding protein |
| *orf4608* | 4276257 | 4277555 | 146.34 | 66.89 | 2.19 | arsenical efflux pump membrane protein ArsB |
| *orf0454* | 445720 | 446388 | 151.65 | 70.99 | 2.14 | acyl-CoA dehydrogenase |
| *orf1706* | 1568017 | 1569267 | 321.39 | 152.02 | 2.11 | hypothetical protein |
| *orf0909* | 833417 | 833998 | 118.83 | 56.42 | 2.11 | MULTISPECIES: hypothetical protein |
| *orf1614* | 1485207 | 1485677 | 540.92 | 259.69 | 2.08 | thiol reductase thioredoxin |
| *orf1281* | 1174210 | 1175412 | 235.47 | 118.37 | 1.99 | Bcr/CflA family multidrug efflux MFS transporter |
| *orf4638* | 4300997 | 4302259 | 537.02 | 276.83 | 1.94 | divalent metal cation transporter |
| *orf2689* | 2475372 | 2475923 | 210.73 | 110.08 | 1.91 | alanine acetyltransferase |
| *orf2756* | 2529030 | 2529431 | 651.44 | 344.22 | 1.89 | manganese transport transcriptional regulator |
| Down-regulating genes | | | | | | |
| *orf4063* | 3715487 | 3716416 | 21.52 | 80.60 | -3.75 | transcriptional regulator |
| *orf403* | 401282 | 402430 | 13.53 | 44.42 | -3.28 | acetylornithine aminotransferase |
| *orf1964* | 1806586 | 1807662 | 132.58 | 418.38 | -3.16 | Cytochrome c oxidase subunit II |
| *orf1965* | 1807692 | 1808219 | 46.24 | 128.94 | -2.79 | cytochrome c oxidase polypeptide I |
| *orf4071* | 3721091 | 3721984 | 26.54 | 67.14 | -2.53 | transcriptional regulator |
| *orf1967* | 1809562 | 1810191 | 75.03 | 183.22 | -2.44 | cytochrome B oxidoreductase |
| *orf4162* | 3823116 | 3824513 | 424.08 | 1035.48 | -2.44 | hypothetical protein |
| *orf1966* | 1808255 | 1809562 | 189.14 | 461.10 | -2.44 | cytochrome ubiquinol oxidase subunit I |
| *orf1963* | 1805597 | 1806487 | 149.96 | 355.21 | -2.37 | protoheme IX farnesyltransferase |
| *orf3460* | 3145930 | 3147309 | 75.82 | 177.74 | -2.34 | argininosuccinate lyase |
| *orf4497* | 4170087 | 4170725 | 63.45 | 140.12 | -2.21 | hypothetical protein |
| *orf4163* | 3824544 | 3825233 | 167.43 | 352.65 | -2.11 | zinc ribbon domain-containing protein |
| *orf2041* | 1871044 | 1874253 | 5899.63 | 12280.08 | -2.08 | carbamoyl phosphate synthase large subunit |
| *orf2810* | 2578796 | 2579503 | 99.40 | 201.36 | -2.03 | bacillithiol biosynthesis deacetylase BshB1 |
| *orf2042* | 1874250 | 1875023 | 618.05 | 1219.85 | -1.97 | dihydroorotate dehydrogenase electron transfer subunit |
| *orf196* | 189572 | 190261 | 614.22 | 1192.22 | -1.94 | DNA-binding response regulator |
| *orf2043* | 1875020 | 1875958 | 492.36 | 936.45 | -1.90 | dihydroorotate dehydrogenase B catalytic subunit |
| *orf193* | 186371 | 186982 | 334.15 | 626.66 | -1.88 | hypothetical protein |

^p^Represented that the gene was located on plasmid p25.
