# Supplementary material for: An operon consisting of a P-type ATPase gene and a transcriptional regulator gene responsible for cadmium resistances in Bacillus vietamensis 151–6 and Bacillus marisflavi 151–25
Source: BMC Microbiol. 2020 Jan 21;20:18. doi: 10.1186/s12866-020-1705-2 (PMC6975044; doi:10.1186/s12866-020-1705-2)
Supplement: Supplementary file 5 — Additional file 5: Table S5. Partial up-regulating genes (fold change> 10) and down-regulating genes (fold change> 20) in the presence of Cd2+ by RNA sequencing for 151–6. [file 12866_2020_1705_MOESM5_ESM.docx]

**Table S5.** Partial up-regulating genes (fold change>10) and down-regulating genes (fold change>20) in the presence of Cd^2+^ by RNA sequencing for 151-6.

| Gene | Location | | Reads count | | Fold change (Cd/CK) | NR annotation |
| --- | --- | --- | --- | --- | --- | --- |
|  | Start | End | Cd | CK |  |  |
| Up-regulating genes | | | | | | |
| *orf4108* | 3741422 | 3741952 | 594.06 | 1.59 | 372.96 | MULTISPECIES: thiol-disulfide oxidoreductase |
| *orf4109* | 3742073 | 3742675 | 762.21 | 3.92 | 194.33 | cytochrome C biogenesis protein CcdA |
| *orf4088* | 3726588 | 3726953 | 177.21 | 1.68 | 105.62 | transcriptional regulator |
| *orf4087* | 3723960 | 3726575 | 7227.01 | 151.22 | 47.79 | ATPase |
| *orf3821* | 3457540 | 3458541 | 77.18 | 2.24 | 34.39 | daunorubicin resistance protein DrrA family ABC transporter ATP-binding |
| *orf3820* | 3456734 | 3457540 | 74.59 | 3.51 | 21.26 | GntR family transcriptional regulator |
| *orf4068* | 3702895 | 3703719 | 775.08 | 39.37 | 19.69 | prolipoprotein diacylglyceryl transferase |
| *orf1313* | 1192873 | 1193724 | 4674.94 | 251.22 | 18.61 | iron transporter FeoB |
| *orf0798* | 774184 | 774606 | 10.57 | 0.61 | 17.44 | ADP-ribose pyrophosphatase |
| *orf4106* | 3740119 | 3740538 | 960.73 | 73.31 | 13.10 | disulfide bond formation protein B |
| *orf1312* | 1192334 | 1192870 | 1406.07 | 111.18 | 12.65 | NA |
| *orf4107* | 3740539 | 3741201 | 3209.45 | 263.75 | 12.17 | Protein-disulfide isomerase |
| *orf0740* | 730452 | 731783 | 17657.02 | 1673.96 | 10.55 | hypothetical protein |
| Down-regulating genes | | | | | | |
| *orf4823* | 4433523 | 4433744 | 5.17 | 246.49 | -47.64 | hypothetical protein |
| *orf2712* | 2482673 | 2482936 | 1.09 | 48.11 | -43.99 | hypothetical protein |
| *orf4825* | 4434602 | 4435144 | 223.44 | 8414.62 | -37.66 | cell wall hydrolase |
| *orf4824* | 4433948 | 4434580 | 456.52 | 15748.71 | -34.50 | muramoylpentapeptide carboxypeptidase |
| *orf4827* | 4435548 | 4436507 | 31.74 | 1071.74 | -33.77 | nucleoside hydrolase |
| *orf4968* | 10268 | 10852 | 1.99 | 64.62 | -32.43 | MULTISPECIES: hypothetical protein |
| *orf4822* | 4432929 | 4433519 | 24.21 | 738.54 | -30.51 | hypothetical protein |
| *orf4826* | 4435176 | 4435328 | 131.45 | 3874.85 | -29.48 | ribonuclease Z |
| *orf1102* | 1007122 | 1007289 | 0.30 | 8.37 | -28.36 | hypothetical protein |
| *orf3232* | 2925190 | 2925573 | 2.58 | 69.81 | -27.09 | hypothetical protein |
| *orf4967* | 9808 | 10275 | 1.00 | 26.58 | -26.58 | MULTISPECIES: hypothetical protein |
| *orf3348* | 3028750 | 3028866 | 0.30 | 7.96 | -26.35 | NA |
| *orf1807* | 1655705 | 1656112 | 1.29 | 32.37 | -25.13 | spore protein |
| *orf4894* | 4497400 | 4497930 | 0.40 | 8.65 | -21.86 | hypothetical protein |
| *orf0890* | 842942 | 843214 | 1.99 | 42.07 | -21.11 | hypothetical protein |
