# Supplementary material for: An operon consisting of a P-type ATPase gene and a transcriptional regulator gene responsible for cadmium resistances in Bacillus vietamensis 151–6 and Bacillus marisflavi 151–25
Source: BMC Microbiol. 2020 Jan 21;20:18. doi: 10.1186/s12866-020-1705-2 (PMC6975044; doi:10.1186/s12866-020-1705-2)
Supplement: Supplementary file 6 — Additional file 6: Figure S1. Growth curve of Bacillus sp. strains (151–6, 151–25, B. subtilis WB600 (BS), B. amyloliquefaciens (BA) and B. licheniformis WX-02 (BL)) at different concentrations of cadmium. [file 12866_2020_1705_MOESM6_ESM.docx]

**
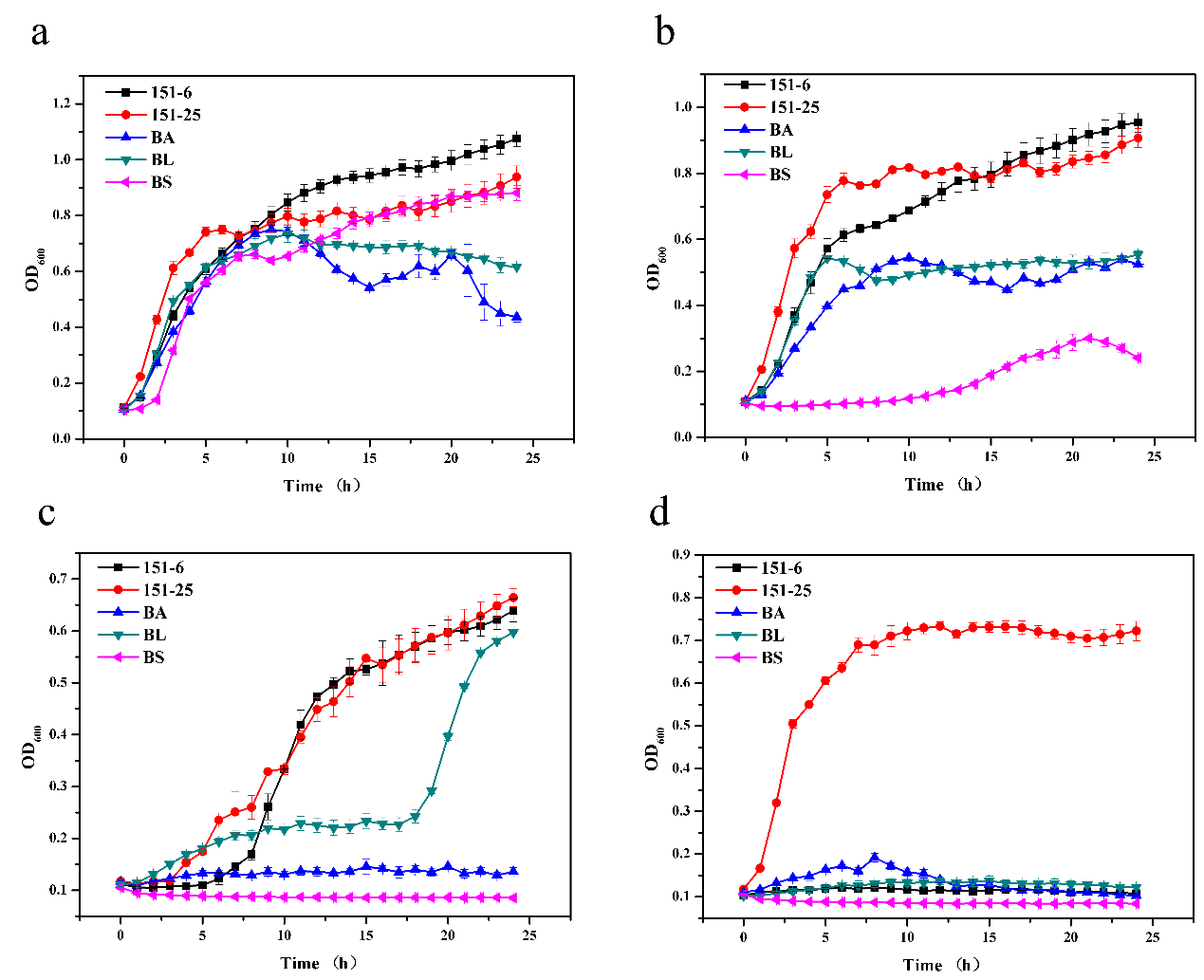
**

**Figure S1.** Growth curve of *Bacillus sp.* strains (151-6, 151-25, *B. subtilis* WB600 (BS), *B. amyloliquefaciens* (BA) and *B. licheniformis* WX-02 (BL)) at different concentrations of cadmium. (a) 0 mM Cd^2+^; (b) 0.1 mM Cd^2+^; (c) 0.3 mM Cd^2+^; (d) 0.5 mM Cd^2+^.
