# Supplementary figures and images for: An operon consisting of a P-type ATPase gene and a transcriptional regulator gene responsible for cadmium resistances in Bacillus vietamensis 151–6 and Bacillus marisflavi 151–25
Source: BMC Microbiol. 2020 Jan 21;20:18. doi: 10.1186/s12866-020-1705-2 (PMC6975044; doi:10.1186/s12866-020-1705-2)

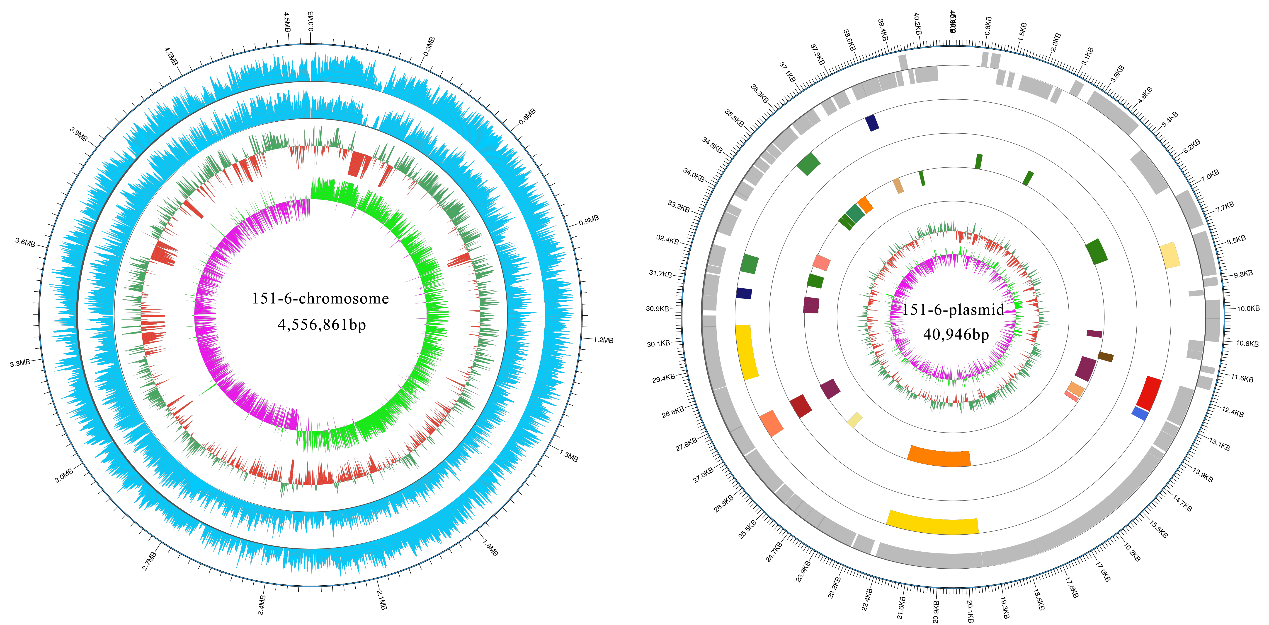


**Figure S2.** Genome map and plasmid map of 151-6.

Supplement: Supplementary file 7 — Additional file 7: Figure S2. Genome map and plasmid map of 151–6. [file 12866_2020_1705_MOESM7_ESM.docx]

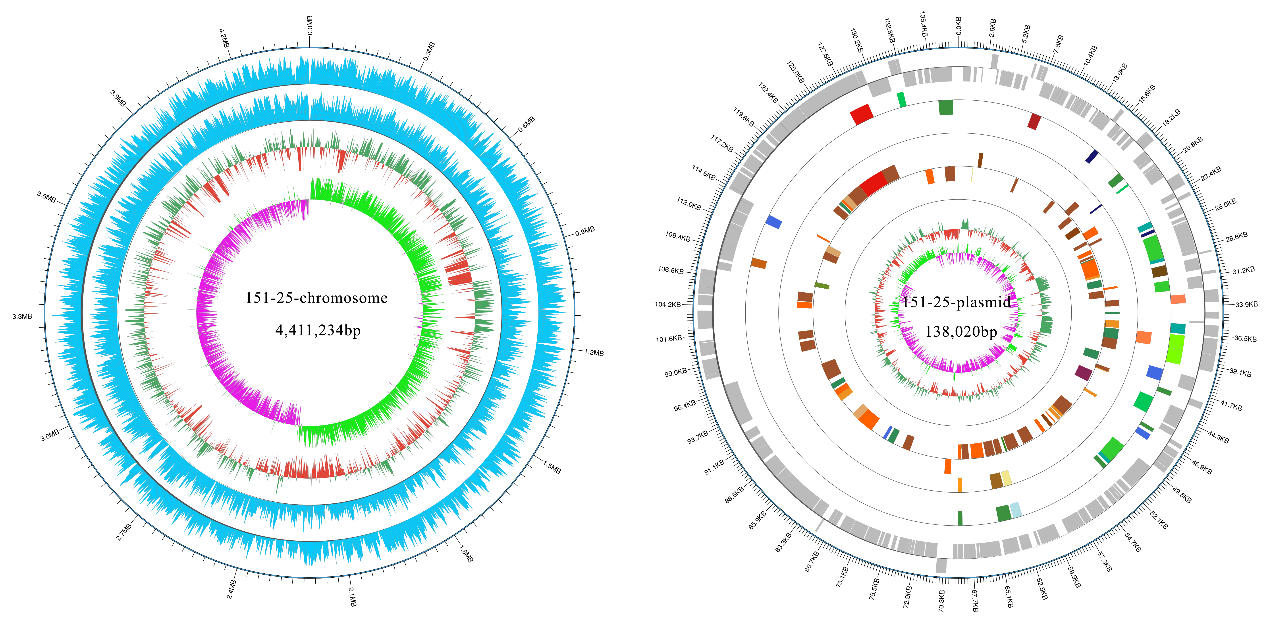


**Figure S3.** Genome map and plasmid map of 151-25.

Supplement: Supplementary file 8 — Additional file 8: Figure S3. Genome map and plasmid map of 151–25. [file 12866_2020_1705_MOESM8_ESM.docx]

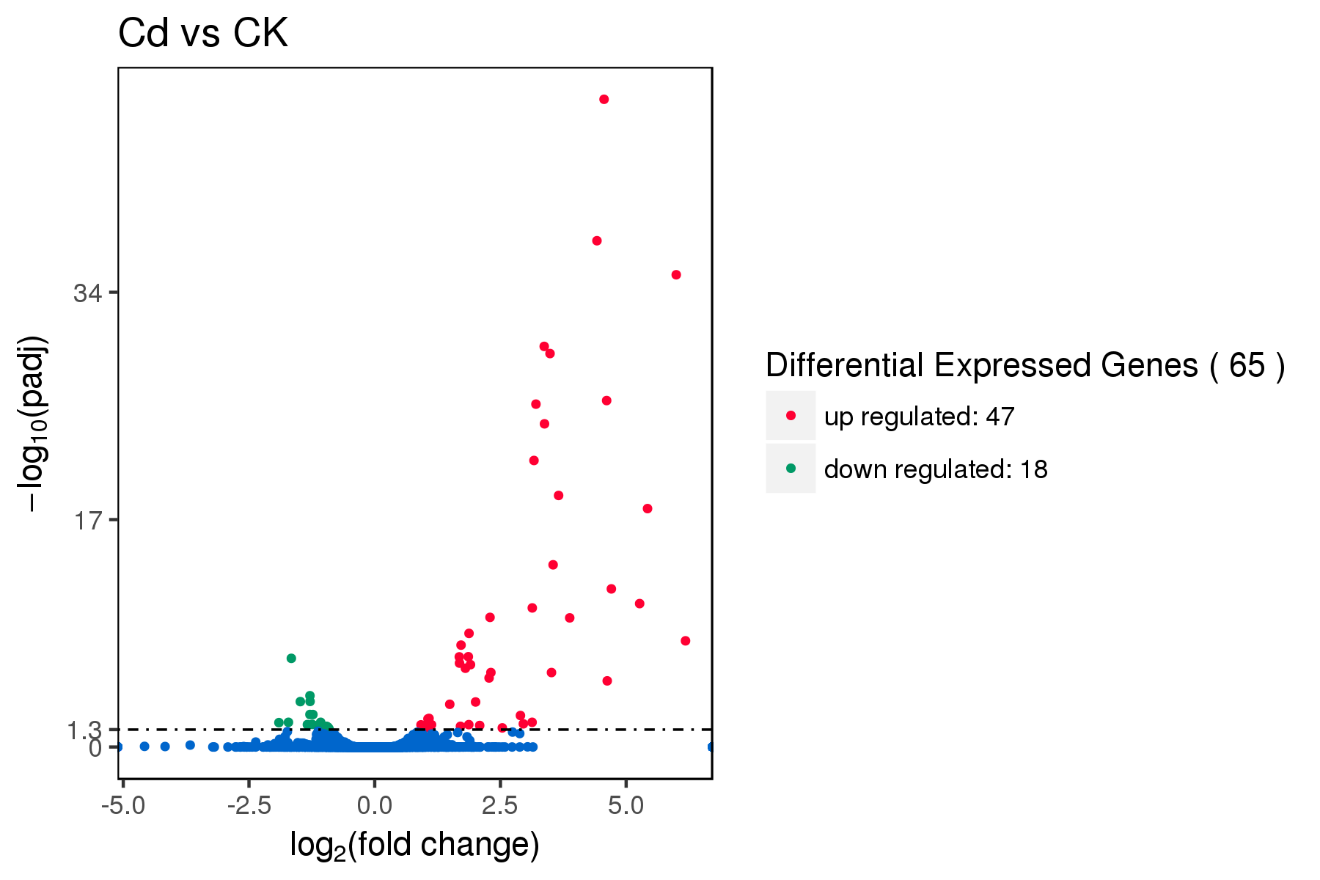


**Figure S6.** Volcano map of differentially expressed genes of 151-25.

Supplement: Supplementary file 11 — Additional file 11: Figure S6. Volcano map of differentially expressed genes of 151–25. [file 12866_2020_1705_MOESM11_ESM.docx]

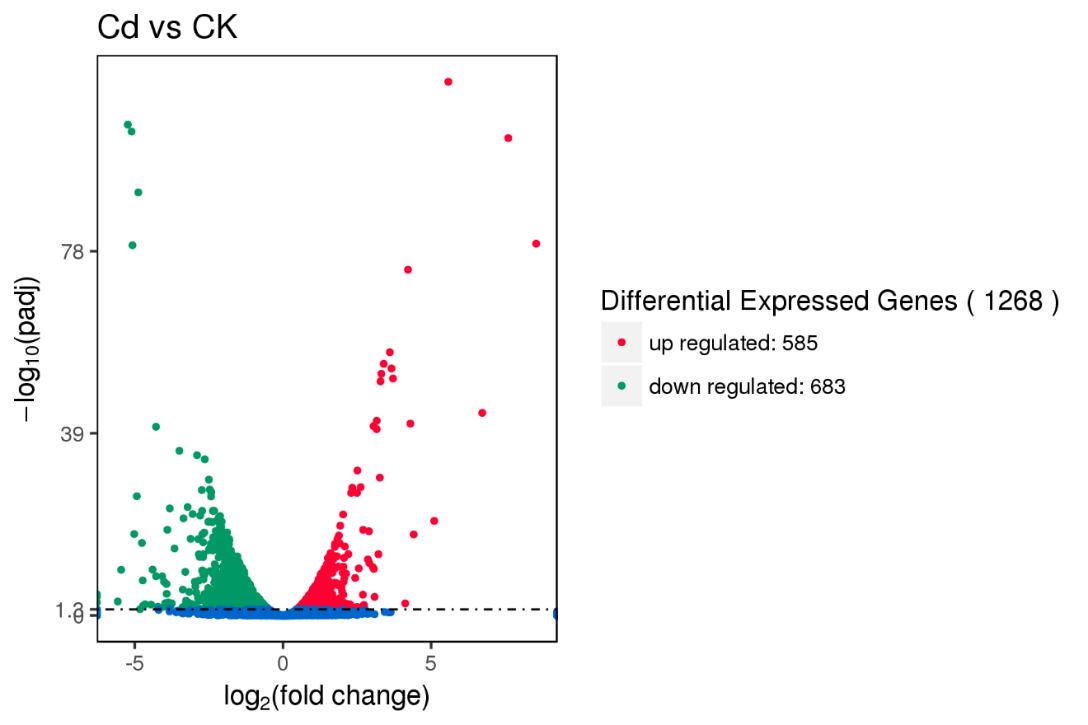


**Figure S8.** Volcano map of differentially expressed genes of 151-6.

Supplement: Supplementary file 13 — Additional file 13: Figure S8. Volcano map of differentially expressed genes of 151–6. [file 12866_2020_1705_MOESM13_ESM.docx]

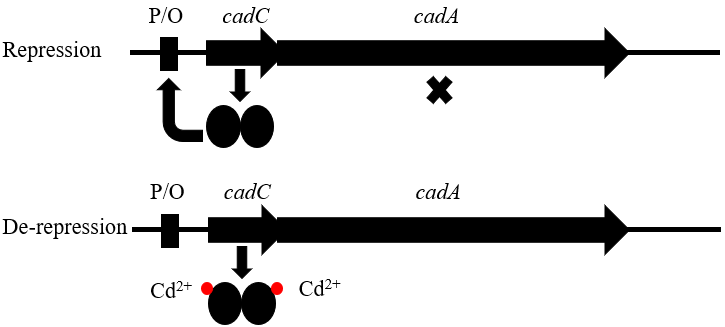


**Figure S12.** The hypothetical *cad* system model of *B. marisflavi* 151-25

Supplement: Supplementary file 17 — Additional file 17: Figure S12. The hypothetical cad system model of B. marisflavi 151–25 [file 12866_2020_1705_MOESM17_ESM.docx]
