# Supplementary material for: An operon consisting of a P-type ATPase gene and a transcriptional regulator gene responsible for cadmium resistances in Bacillus vietamensis 151–6 and Bacillus marisflavi 151–25
Source: BMC Microbiol. 2020 Jan 21;20:18. doi: 10.1186/s12866-020-1705-2 (PMC6975044; doi:10.1186/s12866-020-1705-2)
Supplement: Supplementary file 9 — Additional file 9: Figure S4. Verification of the elimination of the plasmid p6 for 151–6. [file 12866_2020_1705_MOESM9_ESM.docx]

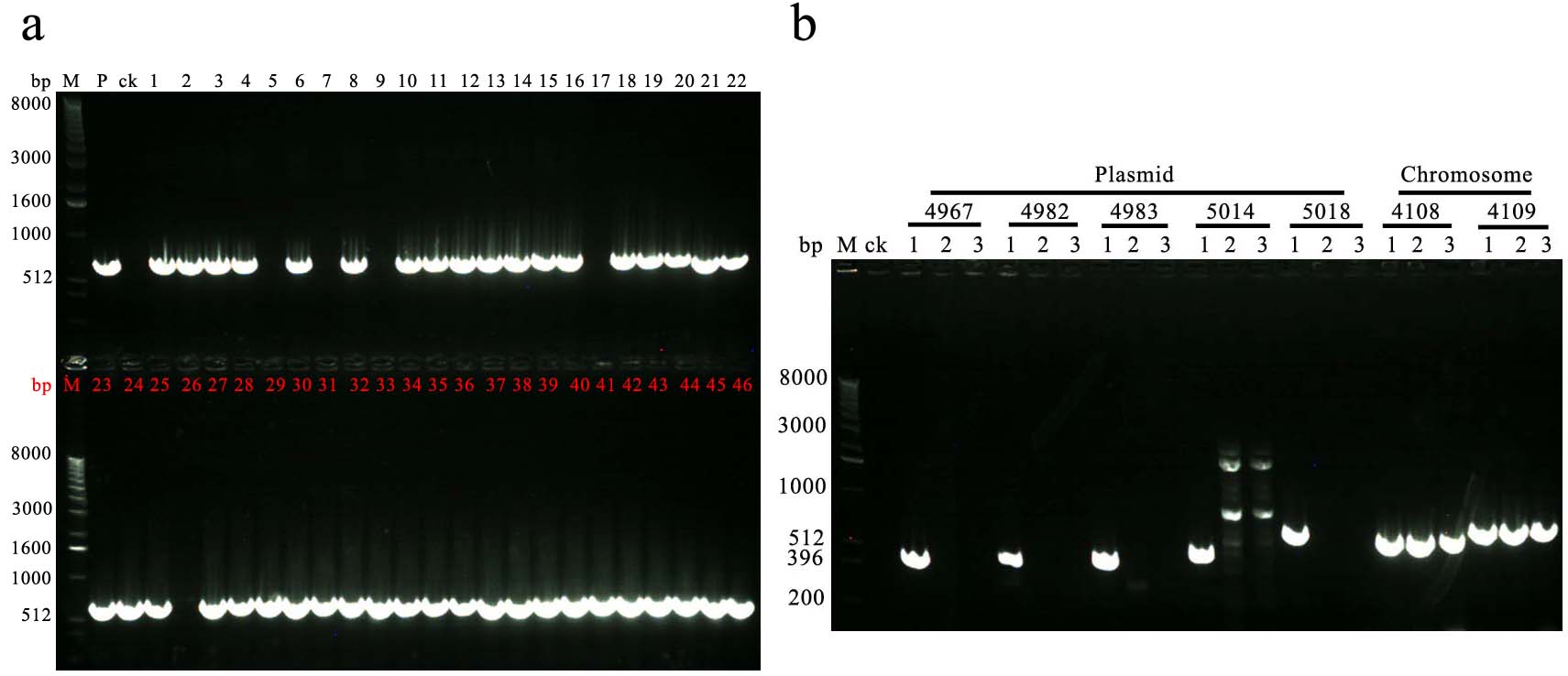


**Figure S4.** Verification of the elimination of the plasmid p6 for 151-6. (a)Selection of elimination of the plasmid p6 by PCR with the primers 4963CDS-F/4963CDS-R (located on plasmid p6). Lanes: M, 1-kb DNA ladder; templates: ck, ddH_2_O; P, 151-6 genome DNA; 1-46: Random selected single colony strain. (b) Identification of elimination of the plasmid p6 by PCR with the primers 4967CDS-F/4967CDS-R, 4982CDS-F/4982CDS-R, 4983CDS-F/4983CDS-R, 5014CDS-F/5014CDS-R, 5018CDS-F/5018CDS-R (located on plasmid p6), 4108CDS-F/4108CDS-R and, 4109CDS-F/4109CDS-R (located on chromosome of 151-6). Lanes: M, 1-kb DNA ladder; templates: ck, ddH_2_O; 1, 151-6 genome DNA; 2: Genome DNA of elimination of the plasmid p25 151-6△5 (marked with the red arrow); 3: Genome DNA of elimination of the plasmid p6 151-6△7(marked with the red arrow).
