# Supplementary material for: An operon consisting of a P-type ATPase gene and a transcriptional regulator gene responsible for cadmium resistances in Bacillus vietamensis 151–6 and Bacillus marisflavi 151–25
Source: BMC Microbiol. 2020 Jan 21;20:18. doi: 10.1186/s12866-020-1705-2 (PMC6975044; doi:10.1186/s12866-020-1705-2)
Supplement: Supplementary file 10 — Additional file 10: Figure S5. Verification of the elimination of the plasmid p25 for 151–25. [file 12866_2020_1705_MOESM10_ESM.docx]

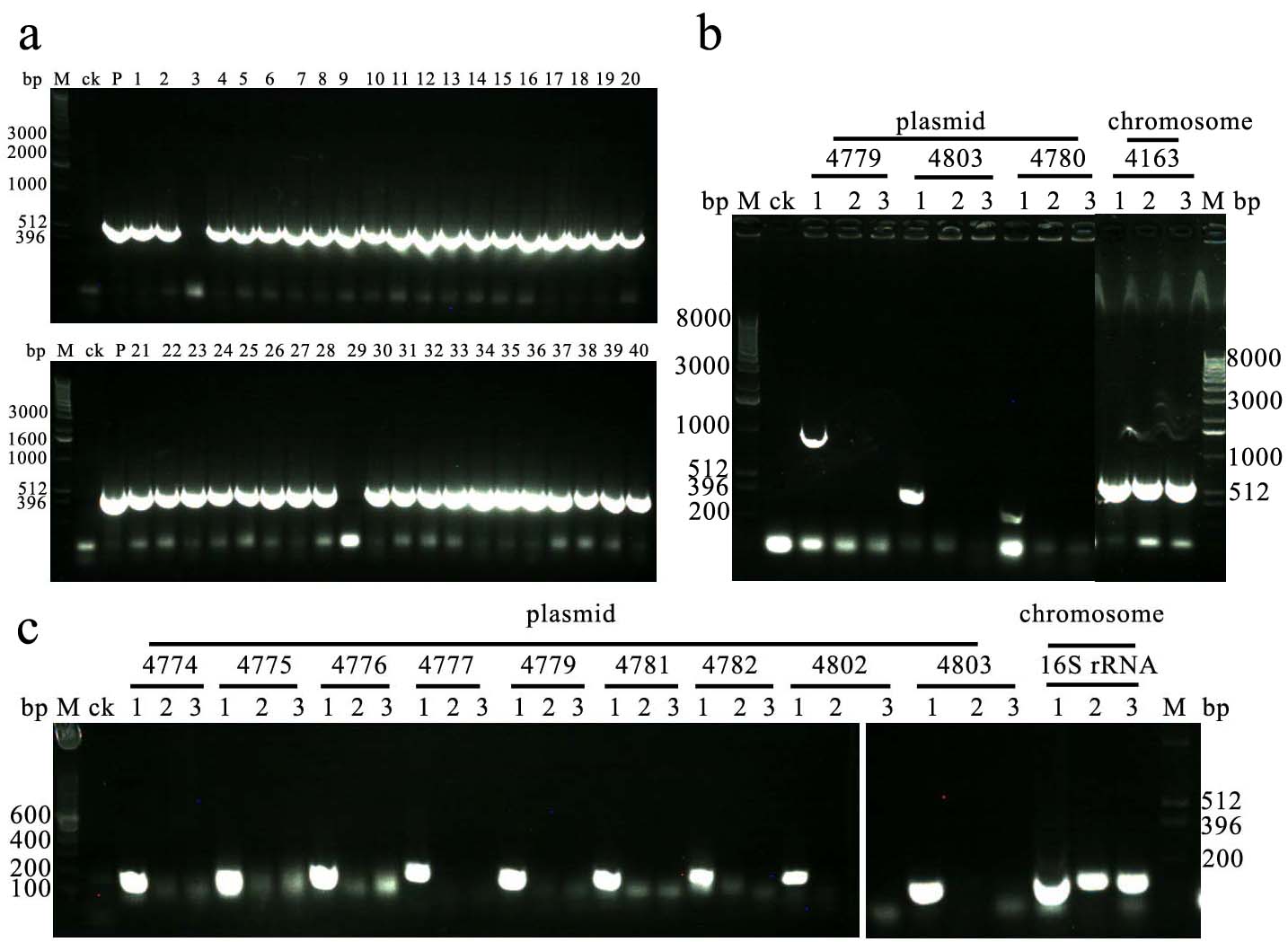


**Figure S5.** Verification of the elimination of the plasmid p25 for 151-25. (a)Selection of elimination of the plasmid p25 by PCR with the primers 4803CDS-F/4803CDS-R (located on plasmid p25). Lanes: M, 1-kb DNA ladder; templates: ck, ddH_2_O; P, 151-25 genome DNA; 1-40: Random selected single colony strain. (b) Identification of elimination of the plasmid p25 by PCR with the primers 4779CDS-F/4779CDS-R, 4803CDS-F/4803CDS-R, 4780CDS-F/4780CDS-R (located on plasmid p25) and 4163CDS-F/4163CDS-R (located on chromosome of 151-25). Lanes: M, 1-kb DNA ladder; templates: ck, ddH_2_O; 1, 151-25 genome DNA; 2: Genome DNA of elimination of the plasmid p25 151-25△3 (marked with the red arrow); 3: Genome DNA of elimination of the plasmid p25 151-25△29(marked with the red arrow). (c) Identification of elimination of the plasmid p25 by PCR with the 151-25 qRT-PCR primers. Lanes: M, 100-bp DNA ladder; templates: ck, ddH_2_O; 1, 151-25 genome DNA; 2: Genome DNA of elimination of the plasmid p25 151-25△3; 3: Genome DNA of elimination of the plasmid p25 151-25△29.
