# Supplementary material for: An operon consisting of a P-type ATPase gene and a transcriptional regulator gene responsible for cadmium resistances in Bacillus vietamensis 151–6 and Bacillus marisflavi 151–25
Source: BMC Microbiol. 2020 Jan 21;20:18. doi: 10.1186/s12866-020-1705-2 (PMC6975044; doi:10.1186/s12866-020-1705-2)
Supplement: Supplementary file 12 — Additional file 12: Figure S7. Transcription analysis of up-regulated genes based on RNA sequencing and examination of Cd-MIC for recombinant B. subtilis containing the operon of 151–25. [file 12866_2020_1705_MOESM12_ESM.docx]

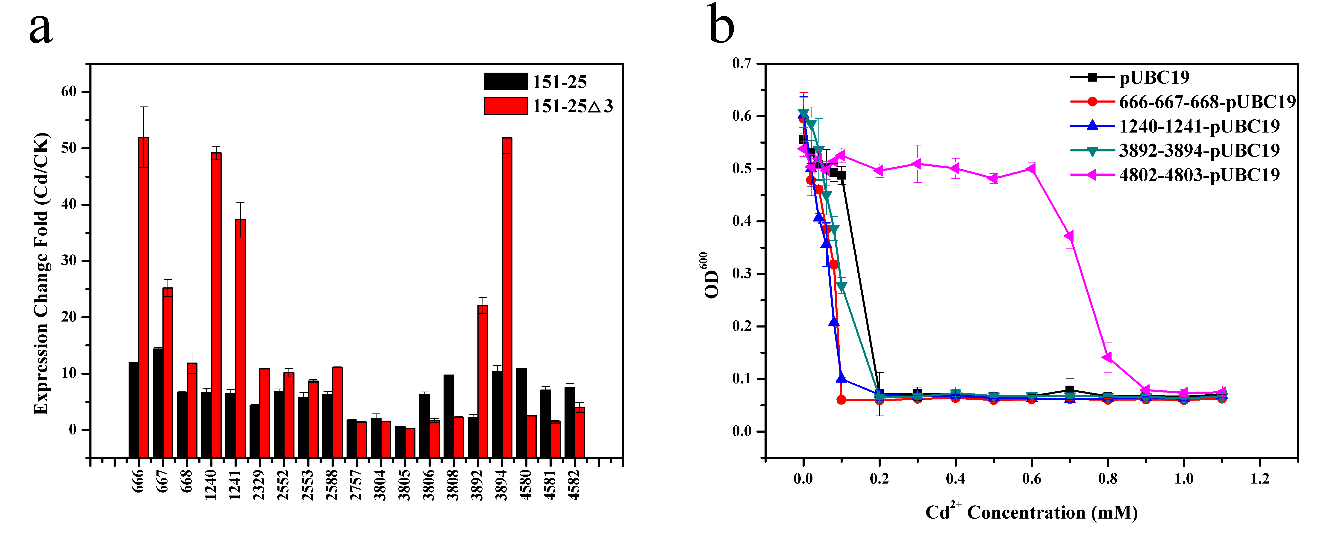


**Figure S7.** Transcription analysis of up-regulated genes based on RNA sequencing and examination of Cd-MIC for recombinant *B. subtilis* containing the operon of 151-25. (a) Transcription analysis of the chromosomal genes orf666, 667, 668, 1240, 1241, 2329, 2552, 2553, 2588, 2757, 3804, 3805, 3806, 3808, 3892, 3894, 4580, 4581 and 4582 in 151-25 cultured with 0.1 mM Cd^2+^ (Cd) in comparison with a culture grown in the absence of Cd^2+^ (CK) using qRT-PCR. Transcript levels of the tested genes were normalized to the 16S rRNA gene. (b) Determination of Cd-MIC for recombinant *B. subtilis* containing the vectors pUBC19 (negative control), 666-667-668-pUBC19, 1240-1241-pUBC19, 3892-2894-pUBC19 and 4802-4803-pUBC19 with varying concentrations of Cd^2+^ (0, 0.02, 0.04, 0.06, 0.08, 0.1, 0.2, 0.3, 0.4, 0.5, 0.6, 0.7, 0.8, 0.9, 1.0 and 1.1 mM).
