# Supplementary material for: An operon consisting of a P-type ATPase gene and a transcriptional regulator gene responsible for cadmium resistances in Bacillus vietamensis 151–6 and Bacillus marisflavi 151–25
Source: BMC Microbiol. 2020 Jan 21;20:18. doi: 10.1186/s12866-020-1705-2 (PMC6975044; doi:10.1186/s12866-020-1705-2)
Supplement: Supplementary file 14 — Additional file 14: Figure S9. Evaluation of Cd-MIC of B2 and C2. B2 and C2 were screened clones in E. coli fosmid library for 151–6. EPI300-T1R was negative control E. coli strain. [file 12866_2020_1705_MOESM14_ESM.docx]

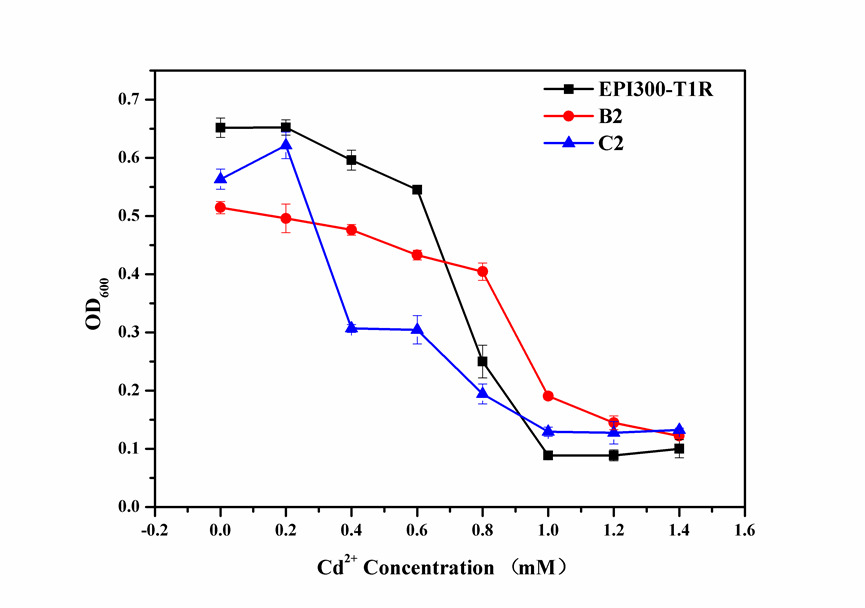


**Figure S9.** Evaluation of Cd-MIC of B2 and C2. B2 and C2 were screened clones in *E. coli* fosmid library for 151-6. EPI300-T1R was negative control *E. coli* strain.
