# Supplementary material for: An operon consisting of a P-type ATPase gene and a transcriptional regulator gene responsible for cadmium resistances in Bacillus vietamensis 151–6 and Bacillus marisflavi 151–25
Source: BMC Microbiol. 2020 Jan 21;20:18. doi: 10.1186/s12866-020-1705-2 (PMC6975044; doi:10.1186/s12866-020-1705-2)
Supplement: Supplementary file 16 — Additional file 16: Figure S11. Alignment of the putative cadc amino acid sequence (4112, 4803) from 151 to 6 and 151–25 versus the CadC from S. aureus. [file 12866_2020_1705_MOESM16_ESM.docx]

**
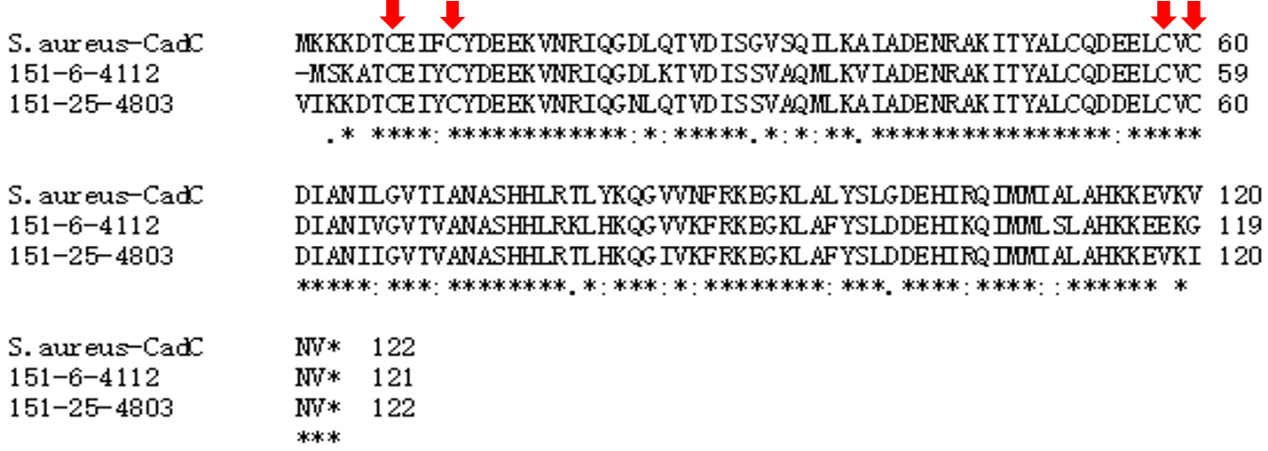
**

**Figure S11.** Alignment of the putative *cadc* amino acid sequence (4112, 4803) from 151-6 and 151-25 versus the CadC from *S. aureus*. Dashes indicate gaps introduced to optimize alignment, asterisk indicate identities, and dots indicate conservative amino acid replacements, semicolons and dots indicate difference and the red arrow represent conserved Cys sites.
